# Chronic *Toxoplasma gondii* Infection Alleviates Experimental Autoimmune Encephalomyelitis by the Immune Regulation Inducing Reduction in IL-17A/Th17 Via Upregulation of SOCS3

**DOI:** 10.1007/s13311-020-00957-9

**Published:** 2020-11-17

**Authors:** Do-Won Ham, Sang-Gyun Kim, Seung-Hwan Seo, Ji-Hun Shin, Sang Hyung Lee, Eun-Hee Shin

**Affiliations:** 1 Department of Tropical Medicine and Parasitology, Seoul National University College of Medicine, and Institute of Endemic Diseases, Seoul, 03080 Republic of Korea; 2 Department of Neurosurgery, SMG-SNU Boramae Medical Center, Seoul National University College of Medicine, Seoul, 07061 Republic of Korea; 3 Seoul National University Bundang Hospital, Seongnam, 13620 Republic of Korea

**Keywords:** *Toxoplasma gondii* infection, EAE, SOCS3, Th17, IL-17A, STAT3, immune regulation, autoimmunity

## Abstract

Experimental autoimmune encephalomyelitis (EAE) is a mouse model of multiple sclerosis (MS), a demyelinating autoimmune disease caused by the infiltration of a harmful autoreactive Th1 and Th17 cells. To mitigate MS, which is impossible to cure with medication only, immunomodulatory interventions that prevent Th17 cell activation are ideal. The objective of the present study was to analyze the effect of *Toxoplasma gondii* infection on the onset of EAE. Our results found that *Toxoplasma gondii* infection in the brain increases SOCS3 expression and decreases the phosphorylation of STAT3, resulting in reducing IL-17A and IL-23, which suppress the differentiation and expansion of pathogenic Th17 cells, an important factor in MS development. These immune responses resulted in a reduction in the clinical scoring of EAE induced by myelin oligodendrocyte glycoprotein 35–55 immunization. In the EAE group with *T. gondii* infection (Tg + EAE group), Th17-related immune responses that exacerbate the onset of EAE were reduced compared to those in the EAE group. This study suggests that the alleviation of EAE after *T. gondii* infection is regulated in a SOCS3/STAT3/IL-17A/blood–brain barrier integrity-dependent manner. Although parasite infection would not be permitted for MS treatment, this study using *T. gondii* infection identified potential targets that contribute to disease attenuation.

**Supplementary Information:**

The online version contains supplementary material available at 10.1007/s13311-020-00957-9.

## Introduction

Multiple sclerosis (MS) is an inflammatory demyelinating disease of the central nervous system (CNS) and the most common inflammatory neurological disease in young adults [5, 21]. The mean age of diagnosis is approximately 30 years with most patients presenting with periodic neurological relapses [21]. One to two decades after onset, many patients with MS enter the progressive phase of the disease, but nevertheless, the underlying cause of this disease remains elusive [21]. Common neurological manifestations of MS include optic neuritis, diplopia, sensory loss, limb weakness, gait ataxia, and cognitive dysfunction [5, 21]. Main causes of MS development are demyelination, wherein the myelin sheath or the oligodendrocyte cell body is destroyed by the inflammatory process with the infiltration of autoreactive Th1 and Th17 cells [5]. Although some medications have been approved by the US FDA to reduce the number of relapses and attenuate the progression of neurological disability, those drugs are only partly effective, and whether they alter the long-term course of MS remains unclear [5, 21]. For successful MS intervention, strategies must comprise dual action on the CNS, such as both immunomodulatory and neuroprotective effects [5]. For example, immune modulation for disease treatment could be realized through interference with antigen presentation, cytokine shifts, the stimulation of immunological tolerance, the induction of suppressor Treg cells, and the suppression of Th17 development [5].

*Toxoplasma gondii* infection is one factor that induces immune modulation in the brain. *T. gondii* is an important opportunistic intracellular parasite and an apicomplexan pathogen of the CNS [6, 7, 20, 22]. In general, the brain is the most commonly affected site, and infection occurs via congenital transmission and subsequent chronic infection [6]. Regarding the relationship between the incidence of MS and *T. gondii* infection in the brain, a recent study revealed a negative association as the rate of *T. gondii* seropositivity was found to be lower in MS patients than in healthy controls (33.9% *vs* 55%, respectively, *p* = 0.007) [10]. Although this study had some limitations such as the assessment of a small study population (specifically 115 patients and 60 age- and sex-matched healthy subjects) and the presence of specific IgG antibodies against *T. gondii*, the authors suggested that further studies were required to establish the protective role of parasitic infections in MS, such as the hypothesized immunomodulatory effects of parasitic infections on autoimmune diseases [10]. In this regard, our previous study demonstrated the favorable effects of immunosuppression induced by *T. gondii* infection on the pathogenesis and progression of Alzheimer’s disease (AD) in Tg2576 AD mice [7]. In addition, we showed that *T. gondii* infection in the brains of C57BL/6 mice induced SOCS1 to reduce detrimental inflammatory immune response, consequently maintaining chronic infection [6]. With respect to autoimmune diseases, it was reported that *T. gondii* infection inhibits the development of lupus-like syndrome in autoimmune (New Zealand Black × New Zealand White) F1 mice, and this study indicated the involvement of Th1-type cytokines in the development of lupus-like nephritis [4]. To date, several studies have suggested that *T. gondii* modulates host immunity during infection [4, 6, 7, 20]. Immune modulation is a selective strategy of *T. gondii* to maintain a life-long chronic infection in the host by regulating immune activation and host cell effector mechanisms. The direction of immune modulation is determined by changes in the expression of cytokines, regulatory factors of immune responses, and factors controlling cytokine expression such as suppressor of cytokine signaling (SOCS) and signal transducer and activator of transcription (STAT), among others [6, 7, 20, 22]. Effector mechanisms that respond to *T. gondii* infection induce IFN-γ–dependent effects that limit parasite replication [6, 12, 20]. However, because IFN-γ signals upregulate inducible nitric oxide synthase, IFN-γ activation in the brain causes tissue injury and neurodegeneration via the production of toxic metabolites, including nitric oxide (NO) [6, 20]. In this case, *T. gondii* modulates host immunity, leading to prolonged parasitic survival without an excessive inflammatory response in the CNS, through increases in SOCS1 and Arg1 and by reductions in phosphorylated STAT1 (P-STAT1) and NO [6]. For the control of inflammatory responses, it is necessary to study the regulation of anti-inflammatory cytokines, including IL-6 (via the classic IL-6 signaling pathway), IL-10, and IL-27, and effector molecules, such as SOCS3 and STAT3 [17, 18, 20, 22]. During cerebral toxoplasmosis, astrocytes and neurons produce IL-27 and inhibit pathological intracerebral Th17 responses, which might prevent over-reactive T-cell responses, and subsequently, immunosuppressive TGF-β signaling in astrocytes is involved in limiting leukocyte infiltration into the CNS [3]. Comprehensively, immune responses in the *T. gondii*–infected brain are changed in a complicated and diverse manner during chronic infection for parasite survival and proliferation in the CNS. In general, because microarray analysis is very effective to expansively review the immune response, we assayed brain samples after *T. gondii* infection. In the present study, we tracked gene expression for 12 weeks after *T. gondii* infection and, as a result, confirmed increases in *SOCS3* and *IL-27* and the reduction in IL-17A. *SOCS3* was remarkably increased by almost 4.0-fold during chronic infection. Since the expression of those genes strongly suggests the possibility of autoimmune disease suppression, we studied the relationship with experimental autoimmune encephalomyelitis (EAE).

EAE, which is the most commonly used rodent model of MS, is extremely useful to understand basic disease pathophysiology and to develop potential treatments for MS [16]. According to the classic paradigm, CD4^+^ T cells, driven by IL-23 and the production of IL-17 (Th17), were found to be required for EAE induction [16]. For disease onset, IL-17A produced by autoantigen-specific CD4^+^ T cells is a key mediator, and these cells are of the Th17 lineage but distinct from Th1/Th2 cells [16]. IL-17A seems to have an early role in the peripheral activation of T cells that later infiltrate the CNS [14]. Accordingly, immunologically based therapies in EAE can be achieved via immune modulators such as immunosuppressants, by altering factors associated with autoimmune responses, and through immunomodulation, which induces balancing of Treg/Th17 cells [1, 13, 17]. Thus, this study aimed to clarify the characteristics of immune modulation mediated by *T. gondii* and identify potential targets that contribute to disease attenuation.

In the present study, we hypothesized that increases in IL-27 and SOCS3 and a decrease in IL-17A in *T. gondii*–infected mouse brains would inhibit the progression of autoimmune diseases like EAE. Our study analyzed immune characteristics in the CNS during chronic *T. gondii* infection and the effect of *T. gondii* infection on the onset of EAE. For this purpose, we investigated the immune environment based on inflammatory cell infiltration and effector T-cell lineages, specifically by assessing Th1, Treg, and Th17 cells. We also investigated the pathological phenomena of encephalitis, demyelination, and blood–brain barrier (BBB) breakdown, and the interactive pathway SOCS3/p-STAT3/IL-17A, which regulates pathogenic Th17 lineage immune responses. Our study provides a new promising approach for disease attenuation through specific immune modulation based on CNS infection by the parasite *T. gondii*.

## Materials and Methods

### Experimental Animals and Ethics Statement

C57BL/6 mice (Orient Bio, Inc., Seongnam, South Korea) at the age of 7 weeks were intraperitoneally injected with *T. gondii* cysts (ME49 strain) and housed in an animal biosafety level 2 environment at the animal facilities of Seoul National University College of Medicine. All animal experiments were approved by the Institutional Animal Care and Use Committee in Seoul National University (IACUC; SNU-110315-5 and SNU-180803-2-2), and animals were maintained in the facility following standards of the Animal Protection Act and the Laboratory Animal Act in Korea. All surgeries were performed under isoflurane anesthesia, and all efforts were made to ensure minimal animal suffering (SNUIBC-R110302-1 and SNUIBC-R180727-1).

### Harvesting *T. gondii* Cysts from Mouse Brains and *T. gondii* Cyst Infection in Experimental Mice

Cysts of *T. gondii* ME49 strain were harvested from the brain tissues of C57BL/6 mice sacrificed 6 weeks after infection. For the microarray analysis of chronically infected mouse brains, mice were intraperitoneally injected with 10 cysts of *T. gondii* and sacrificed at 0 week, 3 weeks, 6 weeks, 9 weeks, 12 weeks, and 24 weeks post-infection (*n* = 3). Brain tissues at each experimental period were collected for microarray analysis. For EAE induction experiments, mice were orally inoculated with 20 cysts of *T. gondii*.

### Microarray Analysis of *T. gondii*–Infected Brains

Total RNA of *T. gondii*–infected brain tissues was pooled (*n* = 3), and microarray analysis was performed by Macrogen, Inc. (Seoul, South Korea), using an Illumina MouseRef-8 v2 Expression BeadChip array (Illumina, Inc., San Diego, CA) according to the manufacturer’s protocol. Arrays were scanned with the Illumina Bead Array Reader Confocal Scanner. Array data export processing and analysis were performed using Illumina GenomeStudio v2011.1 (Gene Expression Module v1.9.0), and the data were analyzed with R v. 2.15.1 statistical software. Hierarchical cluster analysis was performed using Permute Matrix EN software. All heatmaps were generated using Excel spreadsheet software (Microsoft Corporation, Redmond, WA) with conditional formatting. Positive correlations are depicted in red (increased expression), and negative correlations (decreased expression) are depicted in blue. Heatmaps are represented by color scales of the relative minimum (− 4) and maximum (+ 4) values of each factor.

### Induction of EAE

EAE was induced in two experimental groups, specifically EAE and EAE + *T. gondii* (*n* = 6 per group). Mice at 10 weeks after *T. gondii* infection were subcutaneously immunized in both flanks of the back with a 0.2 ml (per mouse) solution of complete Freund’s adjuvant (CFA) emulsion and myelin oligodendrocyte glycoprotein 35–55 (MOG_35–55_) provided from the Hooke Kit MOG35–55/CFA Emulsion PTX (Hooke Laboratories, Lawrence, MA). Then, 0.1 ml of pertussis toxin solution (2 μg/ml) provided by the Hooke Kit was intraperitoneally injected at 1 h and 24 h after MOG35–55/CFA injection. At this time, mice in control and *T. gondii* groups were subcutaneously injected with 0.2 ml phosphate-buffered saline (PBS) on both flanks of the back. Subsequently, 0.2 ml PBS was injected into the mouse intraperitoneally at 1 h and 24 h after the first injection of PBS.

### Disease Severity Grades for EAE

The incidence of EAE was examined every day for 4 weeks according to the manufacturer’s scoring guidelines after immunization with the MOG35–55/CFA emulsion (Table 1, supplementary data). In our study, the disease severity was scored on a scale of 0 to 5. The stage of disease was recorded based on the onset/peak/recovery for each individual mouse.

### Anti-MOG_35–55_ IgG Quantification

The blood of C57BL/6 mice was collected from the orbital sinus under ethyl ether anesthesia (*n* = 6 per group). The collected blood was centrifuged at 1000×*g* for 10 min, and the supernatant (serum) was collected for the detection of anti-MOG IgG levels using a SensoLyte Anti-MOG_35–55_ IgG Quantitative ELISA kit (Fremont, CA). Briefly, the 96-well ELISA plate, which was coated with MOG_35–55_ and provided in the ELISA kit, was incubated with serum samples that were previously diluted 1:5000 with sample dilution buffer provided in the kit, for 1 h at RT. At this time, for the quantification of serum anti-MOG IgG, the titer of the mouse anti-MOG IgG standard, which was provided by the kit, was analyzed based on concentrations of 500 ng/ml, 250 ng/ml, 125 ng/ml, 62.5 ng/ml, 31.25 ng/ml, 15.625 ng/ml, and 7.81 ng/ml, as well as a blank. After washing, the wells were incubated with goat anti-mouse IgG HRP conjugate (1:2000) for 1 h at RT. Finally, colorization of the reaction was achieved by incubating the sample with TMB color substrate solution for 20 min at RT, and then, the reaction was stopped by adding stop solution. Detection was performed using Infinite 200 PRO (Tecan, Männedorf, Switzerland) with i-control microplate reader software (at 450 nm).

### Detection of *T. gondii* in the Brain Using Polymerase Chain Reaction

To confirm *T. gondii* infection, the brain tissues of mice sacrificed after orbital sinus blood sampling were isolated and maintained in PBS on ice. The brain tissue was homogenized using a Dounce glass homogenizer, and genomic DNA was isolated using the DNeasy Blood & Tissue kit (Qiagen, Hilden, Germany) according to the manufacturer’s protocols. The isolated genomic DNA was amplified using conventional polymerase chain reaction (PCR) with 2× Taq PCR Pre-Mix (SolGent Co., Daejeon, South Korea). The PCR was performed based on the following conditions: 95 °C pre-denaturation for 5 min and then 35 cycles (95 °C for 30 s, 57 °C for 30 s, and 72 °C for 30 s) and a final extension at 72 °C for 5 min. The PCR products were confirmed using 1.5% agarose gels containing StaySafe Nucleic Acid Gel Stain (Real Biotech Corporation, Taiwan) in the DNR MiniLumi gel documentation system (DNR Bio-Imaging Systems, Jerusalem, Israel). Primer sequences for the B1 gene are shown in Table 2 (supplementary data).

### Detection of Neuroinflammation Using H&E Staining and Detection of Destroyed Myelin Sheets in the CNS Using Luxol Fast Blue Staining

The brains and spinal cords were isolated from experimental mice and fixed with 4% paraformaldehyde at RT. For further H&E staining, tissues dehydrated with alcohol serial dilutions (70~100%) were embedded in paraffin and sectioned on slides at a thickness of 4 μm. Sectioned tissues were deparaffinized with xylene and hydrated with an alcohol serial dilution (100~70%). After washing for 15 min, the tissues were incubated with Harris hematoxylin solution for 5 min at RT, and then, the tissues were incubated with 1% HCl solution. After washing with tap water, they were incubated with eosin solution for 3 s at RT. After washing, specimens were dehydrated with an alcohol serial dilution (70~100%) and cleared with xylene. After washing, the slides were mounted with coverslips and used to determine neuronal degeneration and inflammation under optical microscopy. For Luxol fast blue (LFB) staining, deparaffinized tissue sections were incubated with LFB stain solution at 56 °C overnight. After washing, tissue sections were incubated with 0.05% lithium carbonate for 20 s. After washing, tissue sections were used to check whether the gray matter was clear and whether the white matter looked clear by optical microscopy. After mounting the slides with coverslips, the tissue sections were observed to assess the destroyed myelin sheets in brains and spinal cords using optical microscopy.

### Quantitative Real-Time PCR

Quantitative real-time PCR was performed on target genes using the CFX96 Real-Time PCR Detection System (Bio-Rad Laboratories, Hercules, CA) and SYBR Green PCR Master Mix (TOPreal qPCR 2X PreMIX; Enzynomics, Daejeon, South Korea). Sequences of primers are shown in Table 2 (supplementary data). Total RNA was isolated from brain tissues using the HiGene BioFACT Total RNA Prep Kit (Ver.2.0) (BioFACT Co., Daejeon, South Korea) according to the manufacturer’s protocols. RNA was reverse-transcribed to cDNA using the Reverse Transcription Master Premix kit (ELPIS Biotech, Daejeon, South Korea). qRT-PCR was performed for the amplification of target genes. Glyceraldehyde 3-phosphate dehydrogenase (GAPDH) expression in each sample was evaluated as an internal control.

### Isolation of Mononuclear Cells in the Brain

Brain tissues were placed in 3.5 ml complete RPMI 1640 Medium (WELGENE, Gyeongsan, South Korea) supplemented with 10% fetal bovine serum (WELGENE) and 1% antibiotic antimycotic solution (WELGENE) and homogenized using a Dounce glass homogenizer (Thomas Scientific, Swedesboro, NJ). The homogenized sample of 3.5 ml was moved to a 15-ml conical tube containing 1.5 ml of 90% Percoll PLUS Centrifugation Media (GE Healthcare, Chicago, IL) to a total volume of 5 ml and a final Percoll concentration of 30%. After vortexing, the sample was moved to a 15-ml conical tube containing 2 ml of 70% Percoll solution overlaid with 30% Percoll on top. This was followed by centrifugation (500×*g*, 18 °C, 30 min). At this stage, mononuclear cells were trapped between the 30% and 70% Percoll, and those cells were collected to a 15-ml conical tube containing 5 ml PBS and centrifuged at 500×*g* for 7 min at 18 °C for washing. The pellets were suspended in PBS and centrifuged at 500×*g* for 10 min at 4 °C for repeated washing. Finally, the pellets were suspended in 500 μl FACS buffer (1% bovine serum albumin (BSA)-PBS) for further FACS analysis. FACS buffer comprised PBS containing 1% BSA (Thermo Scientific Fraction V Bovine Albumin; Thermo Fisher Scientific, Waltham, MA).

### Flow Cytometry to Determine IL-17A Levels in Mononuclear Cells in the Brain

For flow cytometry (FACS), the cells suspended in FACS buffer were permeabilized with Triton X-100 (Sigma-Aldrich, St. Louis, MS). Rat anti-mouse CD16/CD32 antibody (Ab) (BD Pharmingen, Franklin Lakes, NJ) was used to block the Fc receptors, and FITC anti-IL-17A antibody (eBioscience, San Diego, CA) was used to stain IL-17A. The stained cells were analyzed with a FACSCalibur (Becton Dickinson, Franklin Lakes, NJ).

### Western Blotting

To determine the protein levels of SOCS3, Claudin-5, and phosphorylated STAT3, total proteins of the brain tissue were extracted using the M-PER Mammalian Protein Extraction Kit (Pierce Biotechnology, Inc., Waltham, MA), and their concentrations were quantified using a Pierce BCA Protein Assay Kit (Thermo Fisher Scientific). The proteins were separated on 10% SDS polyacrylamide gels for 110 min at 80 V at RT and then transferred to a PVDF membrane for 90 min at 80 V at 4 °C. After blocking with 3% BSA/0.1% TBS-T at RT, the membrane was incubated with each of anti-SOCS3 (Abcam, Cambridge, UK), anti-Claudin-5 (Invitrogen, Carlsbad, CA), anti-STAT3 (Santa Cruz Biotechnology, Dallas, TX), anti-pSTAT3 (Abcam, Cambridge, UK), and anti-β-actin (Santa Cruz Biotechnology) at 4 °C overnight. The m-IgGκ BP-HRP (Santa Cruz Biotechnology) or HRP–goat anti-rabbit IgG (Thermo Fisher Scientific) antibody was used as a secondary antibody. After washing, the membrane was incubated with Pierce ECL Western Blotting Substrate (Thermo Fisher Scientific) for 30 s. The signal was detected with an Amersham Imager 600 (GE Healthcare, Pittsburgh, PA), and the signal intensity was evaluated using ImageJ software.

### Immunofluorescence

Paraffin-embedded brain and spinal cord tissues were sectioned to 4 μm and dried at 60 °C for 1 h (*n* = 6 per group). The sections were soaked in xylene for 10 min and subsequently in an alcohol dilution series (100~30%) for rehydration. After washing, the sections soaked in sodium citrate buffer containing sodium citrate tribasic dehydrate (Sigma-Aldrich, Inc.) were boiled in a microwave for antigen retrieval. After washing with PBS and permeabilization with Triton X-100 (Sigma-Aldrich, Inc.), the sections were blocked with 1% BSA/PBS for 30 min at RT. For immunostaining, the sections were incubated with fluorescence-conjugated anti-IL-17A Ab (FITC, eBioscience). Other targets were co-stained with primary Abs and fluorescence-conjugated secondary Abs; anti-SOCS3 Ab (Abcam, Cambridge, UK), anti-Claudin-5 (Invitrogen), anti-pSTAT3 (Abcam, Cambridge, UK), anti-IL-27 (MyBioSource, San Diego, CA), anti-CD25 (Cell Signaling, Beverly, MA), anti-GM-CSF (Invitrogen), anti-GFAP (Abcam, Cambridge, UK), anti-Iba-1 (Abcam, Cambridge, UK), anti-CD8 (Invitrogen), anti-CD8 antibody (BD Bioscience), anti-CD3 (Invitrogen), and anti-CD4 antibody (Invitrogen) were used as primary Abs, and Alexa Fluor 488–conjugated donkey anti-rabbit IgG (Invitrogen), Alexa Fluor 647–conjugated donkey anti-rabbit IgG (Jackson ImmunoResearch, Baltimore, MD), or Alexa Fluor 647–conjugated rabbit anti-goat IgG ab (Invitrogen) was used as a secondary Ab. After washing with 0.05% PBS-T, the sections were stained with DAPI in the dark for 5 min at RT, mounted using mounting buffer containing glycerol (Sigma-Aldrich, Inc.), and covered with coverslips. The signals were observed using a fluorescence microscope. Fluorescence intensity (FI) of each signal was quantified (arbitrary units, AU) using ImageJ program (*n* = 6 per group).

### Statistical Analysis

All statistical analyses were performed using GraphPad Prism 5 software (GraphPad, La Jolla, CA). Data are presented as the mean ± standard deviation. Analyses of data were performed based on the Kruskal–Wallis test followed by Dunn’s multiple-comparison test to assess the differences between experimental groups; an asterisk (*) indicates a significant difference compared to the control (*p* < 0.05), and a dagger (†) indicates significant differences between experimental groups (*p* < 0.05). Differences were considered significant when *p* values were < 0.05.

## Results

### *T. gondii* Infection Suppresses the Clinical Manifestation of EAE

**Fig. 1:**
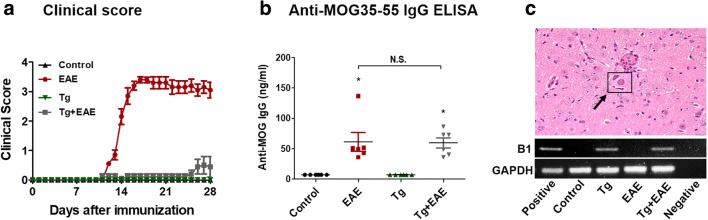
Establishment of experimental autoimmune encephalomyelitis (EAE) model based on MOG_35–55_ immunization, and *Toxoplasma gondii* infection in the brain. (**a**) Clinical scores of EAE in each group. (**b**) EAE induction evaluated based on the elevated anti-MOG IgG titers. (**c**) Morphology of *T. gondii* cysts in the brain and presence of the *T. gondii* B1 gene. Scale bar indicates 100 μm. Data are presented as the mean ± SD of six mice. Asterisk indicates significant difference compared to controls.

EAE induced by MOG_35–55_ immunization can be confirmed by clinical signs scored according to five stages (Supplementary Table 1). Among experimental groups, mice in the EAE group showed clinical scores indicative of approximately stage 3.5 (Fig. 1a). The clinical scoring in the Tg + EAE group was decreased to stage 0.5 (Fig. 1a). To determine the success for the immune triggering of EAE induction after starting MOG_35–55_ immunization, anti-MOG IgG levels were examined by ELISA, and the results showed that the MOG immunization was a success in Tg + EAE groups as well as in the EAE group (Fig. 1b). The results confirming the simultaneous detection of the *T. gondii* B1 gene and the existence of cysts by H&E staining indicated the success of *T. gondii* infection in Tg and Tg + EAE groups (Fig. 1c). Our results showed that the difference in clinical scoring between EAE and Tg + EAE groups was obviously dependent on *T. gondii* infection.

### Microarray Analysis of Brains Chronically Infected with *T. gondii*

**Fig. 2:**
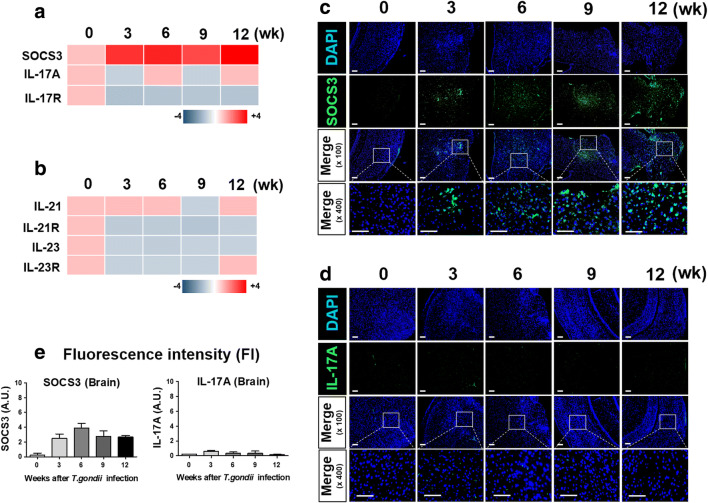
Kinetics of Th17-related molecules in the infected mouse brain. The brain tissues were investigated for Th17 differentiation and secretory factors using microarray analysis. (**a**) Expression kinetics of *SOCS3* and *IL-17A*. (**b**) Expression kinetics of genes encoding secretory factors (IL-21 and IL-23) increased after Th17 cell differentiation. (**c**) Immunofluorescence for SOCS3 expression in *Toxoplasma gondii*–infected brain tissues. (**d**) Immunofluorescence for IL-17A expression in *T. gondii*–infected brain tissues. Scale bar indicates 100 μm. (**e**) Quantification of fluorescence intensity (FI) using ImageJ software.

Given the observed *T. gondii* infection–medicated decrease in EAE clinical scoring in the Tg + EAE group, we investigated changes in gene expression of molecules related with autoimmune disease in brain tissues during *T. gondii* infection (Fig. 2). *SOCS3*, *IL-17A*, and IL-17A receptor A (*IL-17RA*), as markers of the inhibition of Th17 differentiation, and *IL-21*, *IL-23*, and their receptors (*IL-21R* and *IL-23R*) encoding factors that are secreted after Th17 cell differentiation were examined at 3 weeks, 6 weeks, 9 weeks, and 12 weeks after *T. gondii* infection (Fig. 2a, b). Changes in the expression of these genetic markers clearly showed an increase in *SOCS3*, which inhibits Th17 cell differentiation, and a decrease in *IL-17A* and *IL-17RA*, encoding autocrine initiators of Th17 cell differentiation (Fig. 2a). Similarly, the suppression of Th17 differentiation based on the simultaneous reduction of *IL-21* and *IL-23* in the brain after *T. gondii* infection was shown (Fig. 2b). These results were also confirmed based on the protein expression of SOCS3 and IL-17A (Fig. 2c, d). Moreover, the changes in protein expression were also interpreted by the FI, and the results showed that expression of SOCS3 was highly maintained in *T. gondii*–infected brain during the 12 weeks of infection (Fig. 2e). However, *T. gondii* infection did not affect IL-17A expression. This strongly suggests that the immune response induced in the brain after *T. gondii* infection tends to inhibit the differentiation and activation of Th17 cells.

### Distribution of SOCS-3 and P-STAT–Expressing Cells in *T. gondii* Infection and EAE Model

**Fig. 3:**
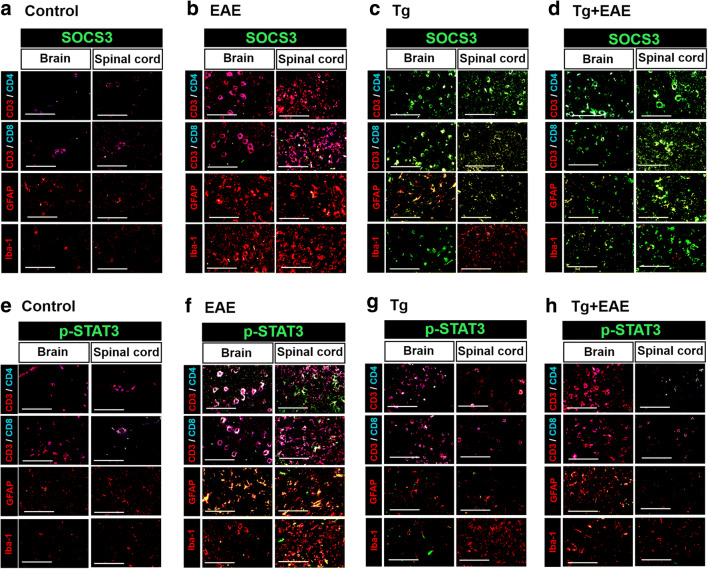
Distribution of SOCS-3 and p-STAT–expressing cells in *T. gondii*–infected brain and spinal cord. CD4^+^/CD3^+^ T cells, CD8^+^/CD3^+^ T cells, astrocytes (GFAP), and microglia (Iba-1) were analyzed for SOCS-3 and p-STAT expression. Fluorescence colors used to stain each cell are as follows: CD3^+^ (red), CD4^+^ (blue), CD8^+^ (blue), SOCS3 (green), GFAP (red), Iba-1 (red), CD3^+^/CD4^+^ (pink), SOCS3/GFAP (yellow), SOCS3/Iba-1 (yellow), CD3^+^/CD4^+^/SOCS3 (white), p-STAT3/GFAP (yellow), p-STAT3/Iba-1 (yellow), and CD3^+^/CD4^+^/p-STAT3 (white). (**a**–**d**) SOCS3-expressing cells in the brain and spinal cord in the control, EAE, Tg, and Tg + EAE groups. (**e**–**h**) p-STAT3–expressing cells in the brain and spinal cord in the control, EAE, Tg, and Tg + EAE groups. Scale bar indicates 100 μm.

To investigate the distribution of SOCS-3 and p-STAT3–expressing cells in the brain and spinal cord, we performed immunofluorescence experiments in the control, EAE, Tg, and Tg + EAE groups (Fig. 3). Cell types and the related markers investigated for SOCS3 and p-STAT3 expression levels were as follows: CD4^+^ T cells (CD4^+^/CD3^+^), CD8^+^ T cells (CD8^+^/CD3^+^), microglia (Iba-1), and astrocytes (GFAP). The mixed colors seen in this figure for cells with expression of SOCS3 (Fig. 3a–d) and p-STAT3 (Fig. 3e–h) were as follows: CD3^+^/CD4^+^/SOCS3 (white), CD3^+^/CD4^+^/p-STAT3 (white), CD3^+^/CD8^+^/SOCS3 (white), CD3^+^/CD8^+^/p-STAT3 (white), SOCS3/GFAP (yellow), p-STAT3/GFAP (yellow), SOCS3/Iba-1 (yellow), and p-STAT3/Iba-1 (yellow). Our data showed that the main expression cells of SOCS3 were as follows: astrocytes in the control group, CD8^+^ T cells and astrocytes in the EAE group, CD4^+^ and CD8^+^ T cells and astrocytes in the Tg group, and CD4^+^ and CD8^+^ T cells and astrocytes in the Tg + EAE group (Fig. 3a–d). Furthermore, the main expression cells of p-STAT3 were as follows: CD8^+^ T cells in the control group, CD4^+^ and CD8^+^ T cells and astrocytes in the EAE group, CD4^+^ and CD8^+^ T cells in the Tg group, and CD8^+^ T cells and astrocytes in the Tg + EAE group (Fig. 3e–h). As a result, the expression of SOCS3 is relatively higher in the Tg and Tg + EAE groups than in the EAE group, and the expression of p-STAT3 is relatively higher in the EAE group than in the Tg and Tg + EAE groups. Accordingly, our data suggests that expression of SOCS3 in the Tg group may provide the early driving force of signal molecule induction for EAE alleviation in the Tg + EAE group.

### Recruitment of T Cells and Resident Immune Cells to the Brain and the Spinal Cord

**Fig. 4:**
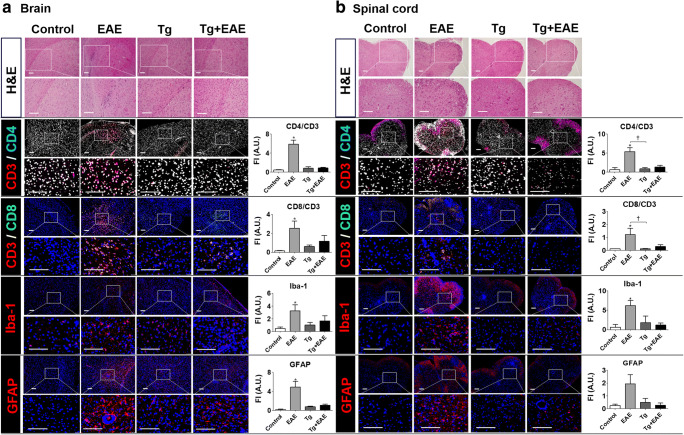
Recruitment of immune cells to the central nervous system (CNS) of experimental autoimmune encephalomyelitis (EAE)–induced and *Toxoplasma gondii*–infected mice. (**a**) Cell recruitment to the brain. (**b**) Cell recruitment to the spinal cord. Hematoxylin and eosin staining. Immunofluorescence staining was performed based on CD3^+^CD4^+^ T cells, CD3^+^CD8^+^ T cells, a microglia marker (Iba-1), and an astrocyte marker (GFAP). The colors of each fluorescent dye are as follows: red (CD3^+^), blue (CD4^+^), and green (CD8^+^). Bar graphs represent the quantified data of fluorescence intensity (FI) in each cell type using ImageJ software. Scale bar indicates 100 μm.

To investigate the reason for the decrease in EAE signs in the Tg + EAE group, we investigated the infiltration and proliferation of CD4^+^ and CD8^+^ T cells and resident inflammatory immune cells (microglia and astrocytes) as an autoimmunity-related cell population (Fig. 4). First, tissue H&E staining of the brain and spinal cord showed a general increase in cell infiltration in the EAE group compared to that in other groups (Fig. 4a, b). Although *T. gondii* infection itself may result in the recruitment and accumulation of resident inflammatory cells, microglia, and lymphocytes responding to infection, the chronically recruited and accumulated inflammatory cells in the Tg-infected group were not significantly different compared to that in control group, indicating that the effect of *T. gondii* infection on CNS pathology is limited. However, the numbers of amassed cells were much higher at the time of EAE induction compared to those with *T. gondii* infection (Fig. 4a, b). To investigate the types of cells that infiltrate into and proliferate in the CNS, cell populations were examined by immunofluorescence staining using antibodies to detect CD4^+^/CD3^+^ T cells, CD8^+^/CD3^+^ T cells, Iba-1 (microglia), and GFAP (astrocytes) (Fig. 4a, b). In EAE-induced mice, we observed an increase in the infiltration and proliferation of CD4^+^ and CD8^+^ T cells, microglia, and astrocytes. However, the infiltration of those cells was reduced in mice in the Tg + EAE group (Fig. 4a, b). These results are well supported by the quantitative analysis of FI. The quantitative data showed that CD4^+^ and CD8^+^ T cells and microglia were significantly increased in the EAE group compared to that in the control in both the brain and spinal cord (*p* < 0.05, Fig. 4a, b). Furthermore, although the quantitative results for the proliferation of astrocytes stained with GFAP also showed an increase in the EAE group, the difference was significant in the brain but not in the spinal cord (*p* < 0.05, Fig. 4a, b). However, the quantitative data showed that cell infiltrations in the Tg + EAE group were reduced to the extent that they were not significantly different compared to those in the control group. Accordingly, it is expected that the reduction in the clinical scoring of EAE in the Tg + EAE group was related to the decrease in pathogenic cell infiltration in the brain and spinal cord.

### *T. gondii* Infection Reduces Neuropathy by Suppressing Demyelination and Maintaining BBB Integrity

**Fig. 5:**
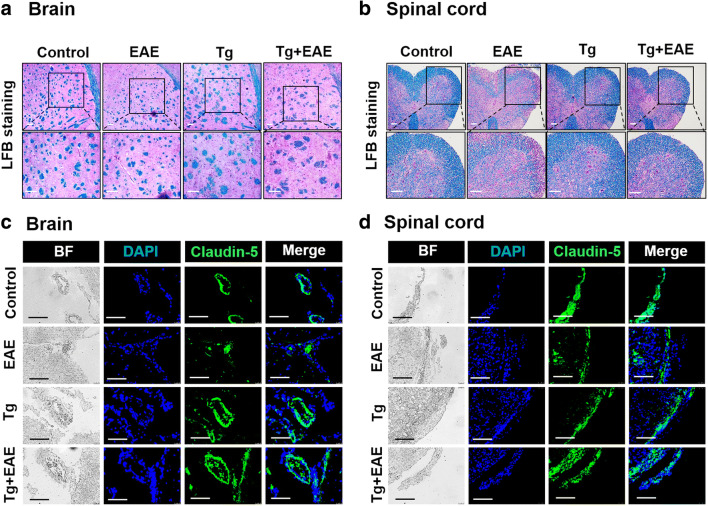
Mitigation of demyelination and BBB breakdown in the central nervous system (CNS) of *Toxoplasma gondii*–infected and experimental autoimmune encephalomyelitis (EAE)–induced mice. (**a**, **b**) Luxol fast blue (LFB) staining to detect myelin in the brain and spinal cord. (**c**, **d**) Immunofluorescence for Claudin-5 as a BBB integrity marker in the brain and spinal cord. Scale bar indicates 100 μm.

The infiltration of inflammatory cells into the CNS results in demyelination of the myelin sheath as a neuropathological result. However, because tissue staining in the Tg + EAE group indicated a decrease in the infiltration of T cells, microglia, and astrocytes, we expected the relief of EAE pathology. To further address this, tissue sections were stained with LFB. *T. gondii* infection increased BBB integrity and decreased damage to the myelin sheath induced by EAE (Fig. 5). LFB is commonly used to detect demyelination in the CNS. Myelin fibers appear blue, neutrophils appear pink, and nerve cells appear purple. As expected, results of LFB staining showed that disruption of the myelin sheath caused by EAE was not prominent in the Tg + EAE group (Fig. 5a, b). The reduction in the disruption of the myelin sheath was clearer in the spinal cord than in the brain, as shown in LFB-stained tissue area. Regarding the reason for this, we speculated that the difference in neuropathy between EAE and Tg + EAE groups might be caused by a difference in BBB integrity (Fig. 5c, d). The degree of immunofluorescent staining for Claudin-5, as a marker of BBB integrity, was most reduced in mice of the EAE group, whereas it was reduced less in the Tg + EAE group (Fig. 5c, d). Considering that there was no difference between Tg and Tg + EAE groups, it seems that the maintenance of BBB integrity in the Tg + EAE group was induced by *T. gondii* infection.

### Immune Environment in the Brain After *T. gondii* Infection and EAE Induction

**Fig. 6:**
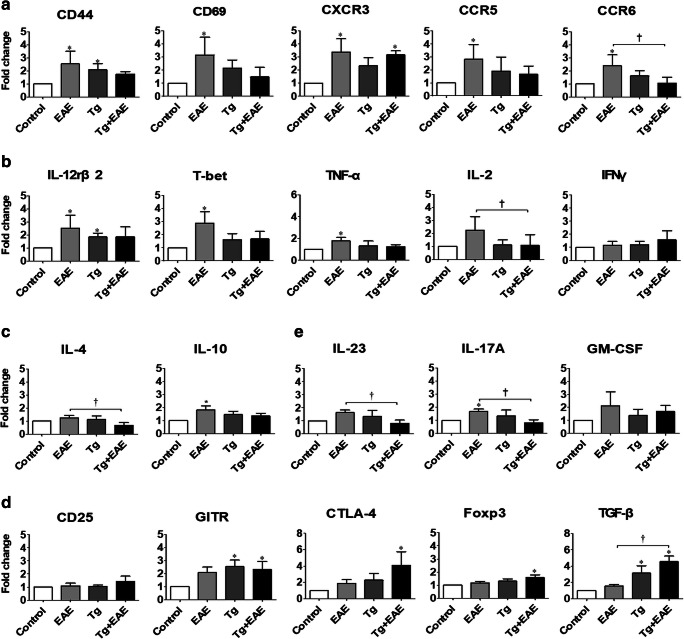
Inflammatory factors and Th17/Treg cell differentiation after *Toxoplasma gondii* infection and experimental autoimmune encephalomyelitis (EAE) induction. (**a**) Gene expression levels of T-cell activation markers and T cell trafficking chemokine receptors. (**b**) Gene expression levels of Th1 cell markers. (**c**) Gene expression levels of Th2 cell markers. (**d**) Gene expression of Treg cell markers. (**e**) Gene expression levels of Th17 lineage cytokines. Data indicate the fold-change value of the target molecule compared to control levels (onefold). Asterisk indicates significant difference compared to controls (*p < 0.05). Dagger indicates significant difference between experimental groups (^†^*p* < 0.05).

The importance of immune cells in EAE pathology is well known. EAE has been considered a Th1 cell– and Th17 cell–mediated disease. Moreover, to mitigate EAE-, Th2-, and Treg-mediated immune responses can be helpful. The immune-triggering effect of EAE in the brain can be characterized by cytokines and chemokines expressed by CD4^+^ T cells, Th17 cells, and Treg cells (Fig. 6). First, T-cell activation markers, specifically CD44 and CD69, and chemokine receptors involved in T-cell trafficking to inflamed peripheral sites of Th1-type inflammation, namely CXCR3, CCR5, and CCR6, were significantly increased in the EAE group (*p* < 0.05, Fig. 6a) but were reduced in the Tg + EAE group compared to levels in the EAE group. Among these, CCR6 levels were significantly reduced (*p* < 0.05, Fig. 6a). Cytokines included in Th1 immune responses, IL-12rβ2, T-bet, TNF-α, and IL-2, were significantly increased in the EAE group, whereas they were decreased in the Tg + EAE group (*p* < 0.05, Fig. 6b). However, the gene expression of IFN-γ was not significantly increased in all the experimental groups (Fig. 6b). Gene expression of cytokines, IL-4 and IL-10, included in Th2 immune responses is shown in Fig. 6c. The gene expression of IL-10 was significantly increased in the EAE group, whereas IL-4 was significantly decreased in the Tg + EAE group although the difference was not much (Fig. 6c). Further, Treg-related molecules such as GITR (glucocorticoid-induced TNFR family-related gene), CTLA4 (cytotoxic T lymphocyte–associated protein), Foxp3, and TGF-β were significantly increased in the Tg + EAE group compared to the control group (*p* < 0.05, Fig. 6d). In contrast, Th17 cell lineage cytokines, namely IL-23 and IL-17A, were increased in the EAE group but were significantly reduced in the Tg + EAE group (*p* < 0.05, Fig. 6e), whereas the change of GM-CSF, a Th17 cell lineage cytokine, was similar to that of IL-23 and IL-17A; however, there was no significant difference among the experimental groups (Fig. 6e). This result strongly suggests that the immune response induced by *T. gondii* infection can change the disease severity of EAE through decreases in Th1 and Th17 cell–polarized immune responses and an increase in Treg cell polarization.

### *T. gondii* Infection Modulates the Immune Response to Increase SOCS3 and IL-27 Expression in the CNS

**Fig. 7:**
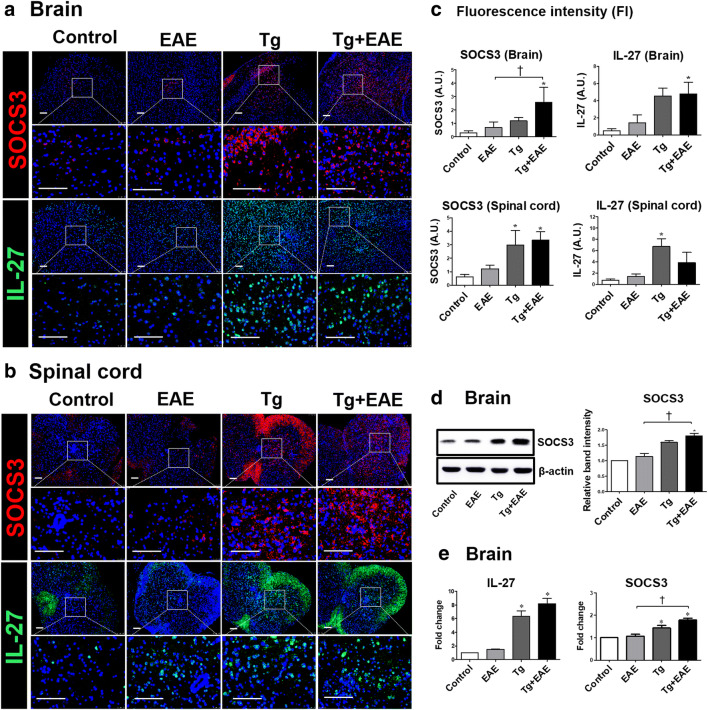
Changes in gene and protein levels of SOCS3 and IL-27 in the brains and spinal cords of *Toxoplasma gondii*–infected and experimental autoimmune encephalomyelitis (EAE)–induced mice. (**a**, **b**) Immunofluorescence staining for SOCS3 and IL-27 expression in the brain and spinal cord. (**c**) Quantification of fluorescence intensity for SOCS3 and IL-27 expression in the brain and spinal cord. (**d**) Western blot image and the relative quantitation of SOCS3 protein expression. (**e**) Gene expression (fold-change) of *IL-27* and *SOCS3* compared to control levels. Scale bar indicates 100 μm. Asterisk indicates significant difference compared to controls (**p < 0.01). Dagger indicates significant difference between experimental groups (^†^*p* < 0.05).

Our microarray results showed that the expression of *SOCS3* was increased by approximately 4.04-fold after *T. gondii* infection, and in addition, immunofluorescence results showed that SOCS3 protein was clearly increased in the brain during chronic *T. gondii* infection (Fig. 2). Since SOCS3 is expressed by immune cells and residential CNS cells, it participates in regulating the immune process within the CNS. In particular, it was predicted that the increase of SOCS3 after *T. gondii* infection would modulate the progression of EAE. As expected, SOCS3 was more highly expressed in the brains and spinal cord of both Tg and Tg + EAE group mice (Fig. 7a–c). To quantify the increase in SOCS3 shown in the immunofluorescence results, the FI was calculated as an AU. SOCS3 in the brain was significantly increased in the Tg + EAE group compared to the control and the EAE group. Similarly, SOCS3 in the spinal cord was significantly increased in the Tg + EAE group as well as the Tg group (*p* < 0.05, Fig. 7c). However, although SOCS3 expression was not induced in the EAE group, it is certain that the increase in SOCS3 was caused by *T. gondii* infection. In addition, IL-27 expression was increased in both the Tg and Tg + EAE groups compared to the control in both the brain and spinal cord; however, a significant difference in IL-27 expression was shown in the Tg + EAE group in the brain and in the Tg group in the spinal cord (*p* < 0.05, Fig. 7c). Given that IL-27 did not increase in the EAE group, these results suggest that *T. gondii* infection induces the increase in SOCS3 and IL-27 in both the brain and spinal cord, and accordingly, it is certain that the increase in SOCS3 and IL-27 in the Tg + EAE group is due to *T. gondii* infection. Further, western blot analysis clearly demonstrated an increase in SOCS3 protein levels after *T. gondii* infection (Fig. 7d). In addition, because SOCS proteins play a role as negative regulators of JAK-STAT signal transduction, the increased gene expression of *IL-27* and *SOCS3* in the Tg + EAE group suggests that STAT3 activation was affected (*p* < 0.05, Fig. 7e). Our results suggest that the increase in SOCS3 might have a role in the inactivation of STAT3 to suppress Th17 cell differentiation, aggravating autoimmune diseases such as EAE.

### *T. gondii* Infection Reduces STAT3 Phosphorylation Through Decreases in IL6/JAK Expression

**Fig. 8:**
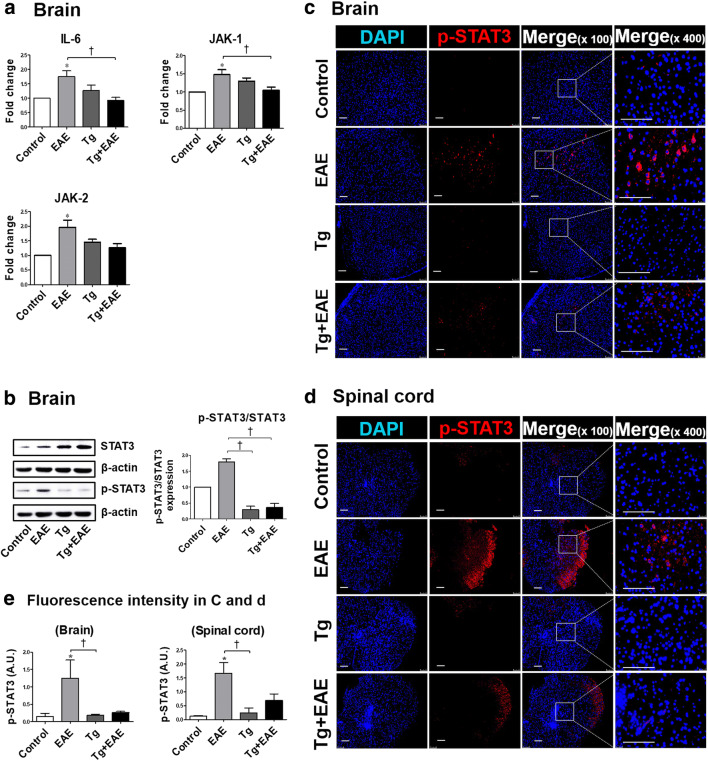
Evaluation of the IL-6/JAK/STAT3 signaling axis in *Toxoplasma gondii*–infected and experimental autoimmune encephalomyelitis (EAE)–induced mouse brains. (**a**) Gene expression of *IL-6* and *JAK-1/2* in the brain. (**b**) Western blot image of STAT3 phosphorylation and ratios of pSTAT3/STAT3. (**c**, **d**) Immunofluorescence staining of p-STAT3 in the brain and spinal cord. (**e**) Quantification of fluorescence intensity for p-STAT3 expression in the brain and spinal cord. Scale bar indicates 100 μm. Asterisk indicates significant difference compared to control levels (**p < 0.05). Dagger indicates significant difference between experimental groups (^†^*p* < 0.05).

In terms of the effect of *T. gondii* infection on the regulation of signaling pathways related to the progression of EAE, the present study targeted the IL-6/JAK/STAT3 axis. For this, we examined whether the inactivation of STAT3 could be regulated by IL-6 and JAK signaling (Fig. 8). The result showed that gene levels of *IL-6*, *JAK-1*, and *JAK-2* in the brain were all decreased in the Tg + EAE group compared to those in the EAE group (Fig. 8a). In addition, the activation of STAT3, as an important molecule of the IL-6/JAK/STAT3 pathway, was evaluated based on its phosphorylation (Fig. 8b). The phosphorylation of STAT3 was decreased in the brain of both the Tg and Tg + EAE groups as shown by western blotting and the pSTAT3/STAT3 ratios (Fig. 8b). Furthermore, the decreased phosphorylation of STAT3 was confirmed by immunofluorescence results (Fig. 8c, d). In addition, quantitative analysis of tissue FI showed that the expression of p-STAT3 was increased only in the EAE group but not in the Tg or the Tg + EAE group (*p* < 0.05, Fig. 8e). These results suggest that the increase in SOCS3 after *T. gondii* infection resulted in a decrease in STAT3 phosphorylation and simultaneously decreased IL-6/JAK/STAT3 pathway activity.

### *T. gondii* Decreases IL-17A Production by Reducing the Transcriptional Regulators RORγt/BATF/RUNX1

**Fig. 9:**
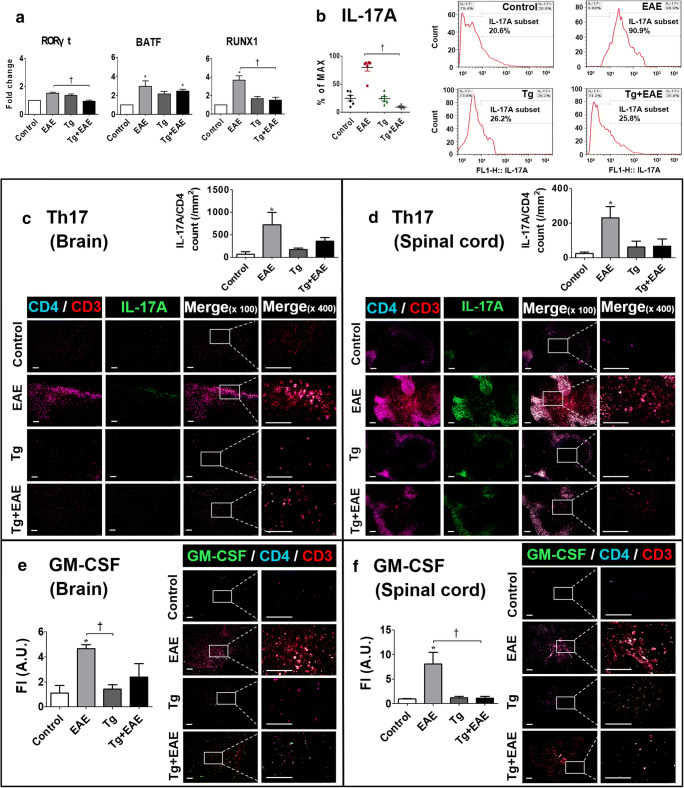
Changes in transcriptional regulators of IL-17A and protein expression of IL-17A. (**a**) Gene expression of *RORγt*/*BATF*/*RUNX1*. (**b**) FACS analysis of IL-17A–expressing brain cells. (**c**, **d**) Distribution of CD3^+^/CD4^+^/IL-17A triple-stained cells in the brain and spinal cord. Triple-stained cells were counted by particle analysis using ImageJ. (**e**, **f**) Distribution of CD3^+^/CD4^+^/GM-CSF triple-stained cells in the brain and spinal cord. The fluorescence intensity of GM-CSF–expressing Th cells in the brain and spinal cord was quantified using ImageJ software. Scale bar indicates 100 μm. Asterisk indicates significant difference compared to control levels (*p < 0.05). Dagger indicates significant difference between experimental groups (^†^*p* < 0.05).

Gene expression of transcriptional regulators including retinoic acid–related orphan nuclear receptor (*RORγt*), basic leucine zipper transcription factor (*BATF*), and Runt-related transcription factor 1 (*Runx1*) was decreased in the brain tissue of both Tg and Tg + EAE groups compared to levels in the EAE group (Fig. 9a). In particular, RORγt, as a master transcription factor involved in IL-17 expression, was significantly decreased in the brain tissue of the Tg + EAE group compared to levels in the EAE and Tg groups (*p* < 0.05, Fig. 9a). IL-17A–expressing cells, as confirmed by FACS analysis, were significantly decreased in Tg and Tg + EAE groups compared to numbers in the EAE group (*p* < 0.05, Fig. 9b). Furthermore, these IL-17A–expressing cells were histologically confirmed as a Th17 cell population by co-staining with CD3^+^, CD4^+^, and IL-17A antibodies in the brain and spinal cord (Fig. 9c, d). The quantification of infiltrating IL-17A^+^/CD4^+^/CD3^+^ Th17 cells confirmed that Th17 cells were significantly increased in the brain and spinal cord of the EAE group, whereas it was decreased in the Tg + EAE group to an extent that was not significantly different compared to the control group (*p* < 0.05, Fig. 8c, d). Similarly, GM-CSF–producing Th cells (Th-GM) were significantly increased in the brain and spinal cord of the EAE group, whereas in the Tg + EAE group, it was reduced to an extent that was not significantly different compared to the control group (*p* < 0.05, Fig. 8e, f). Given that GM-CSF facilitates Th17 cell differentiation by enhancing IL-6 and IL-23, and Th-GM cooperates with Th17 cells to exacerbate the development of inflammation, the decrease in Th-GM exhibited by the present result suggests that the inflammation related to pathogenic Th17 cells was reduced in the Tg + EAE group. Accordingly, our results imply that the decrease in these transcriptional factors controls *IL-17A* transcription and Th17 differentiation, and the decrease in Th-GM reduces inflammation related with pathogenic Th17 cells, which are eventually decreasing the pathogenicity of Th17 cells that cause the infiltration of adaptive and innate immune cells and proliferating CNS resident cells.

### *T. gondii* Infection Prevents BBB Breakdown Caused by EAE

**Fig. 10:**
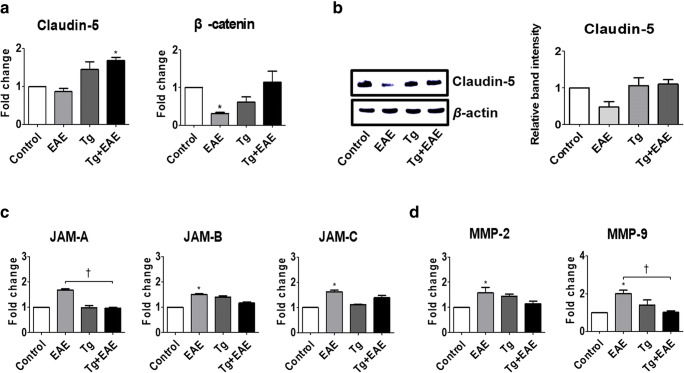
Changes in tight junction–related molecules in *Toxoplasma gondii*–infected and experimental autoimmune encephalomyelitis (EAE)–induced mouse brains. (**a**) Gene expression of *β-catenin* and *Claudin-5*. (**b**) Western blot image and relative band intensity of Claudin-5 expression. (**c**) Gene expression of junctional adhesion molecules (JAMs). (**d**) Gene expression of *MMP-2* and *MMP-9*. Asterisk indicates significant difference compared to control levels (*p < 0.05). Dagger indicates significant difference between experimental groups (^†^*p* < 0.05).

BBB integrity is very important to prevent the gathering of inflammatory cells into inflamed sites during EAE. Tight junction strands serve as a physical barrier to prevent solute transport. Claudin-5 is an integral membrane protein and a component of the tight junction strands. Moreover, β-catenin participates in cell–cell adhesion and gene transcription. The junctional adhesion molecules (JAMs), which are interendothelial junctional molecules, allow circulating leukocytes to enter the CNS by crossing the BBB. As a result of examining the gene expression of Claudin-5 as a gene related to BBB integrity, we found that the expression of Claudin-5 was increased in the Tg and Tg + EAE groups compared to the control and EAE groups, and in particular, Claudin-5 gene in the Tg + EAE group was significantly increased compared to the control group (*p* < 0.05, Fig. 10a). In the EAE mouse model of the present study, gene expression levels of *β-catenin*, which participate in BBB integrity, were decreased compared to control levels; however, they were increased in Tg + EAE mice as compared to levels in the EAE group (Fig. 10a). The protein level of Claudin-5 was also slightly increased in the Tg + EAE group compared to that in the EAE group and became such that there was no difference with the control group (Fig. 10b). In contrast, gene expression levels of *JAM-A*, *JAM-B*, and *JAM-C* as adherent junctional molecules were mostly increased in the EAE group, and in particular, gene expression levels of *JAM-B* and *JAM-C* in the EAE group were significantly increased compared to the control group (*p* < 0.05, Fig. 10c). The gene expression levels of *JAM-A*, *JAM-B*, and *JAM-C* in the Tg and Tg + EAE groups did not increase significantly compared to the control group, which suggests a role in suppressing the recruitment of leukocytes into the inflamed site (Fig. 10c). The significant increase in gene expression of the matrix metalloproteinase family (*MMP-2* and *MMP-9*) in the EAE group suggested the breakdown of the extracellular matrix allowing the transmigration of leukocytes into the CNS parenchyma across the BBB (*p* < 0.05, Fig. 10d). However, the Tg + EAE group mitigated the increase in *MMP-2* and *MMP-9* expression caused by EAE induction (Fig. 10d). These results suggest that *T. gondii* infection prevents the infiltration of inflammatory cells into the CNS parenchyma by preventing BBB breakdown caused by EAE induction.

## Discussion

Our previous studies showed that *T. gondii* regulates immune responses in the CNS after infection in mice [6]. The reason for this is that this organism manipulates the host immune response, which is based on the complicated interplay between host cells and *T. gondii* comprising a multitude of strategies to evade the host immune response [15]. In the brain, immune regulation during chronic *T. gondii* infection induces the inhibition of harmful inflammatory responses through increases in SOCS1 and Arginase1, as well as decreases in pSTAT1 and NO, even with M1 polarization and the activation of microglia and Th1 inflammatory responses [6]. In addition, in the present study, we revealed increases in *SOCS3* and *IL-27*, as well as decreases in *STAT3* and *IL-17A*, through a microarray analysis of *T. gondii*–infected mouse brains. Based on the role of SOCS proteins as immunomodulators involved in different diseases, *T. gondii*-mediated increases in the expression of SOCS1 and SOCS3 in the brain have the potential to impact cerebral immune responses including inflammatory cytokine and chemokine production, the activation of microglia and astrocytes, inflammatory cell infiltration, and autoimmunity [2]. To date, an evaluation of the relationships between *T. gondii* infection and neurodegenerative disease has suggested a favorable effect of *T. gondii* infection in AD mice; however, the relationship between *T. gondii* infection and autoimmune disease was poorly understood [7]. In this regard, we prepared an EAE mouse model using MOG_35–55_ immunization [16]. As a typical immunogenic synthetic peptide, MOG_35–55_ induces a chronic form of EAE in mice that is characterized by mononuclear inflammatory infiltration, demyelination, and spinal cord lesions, which lead to a gradual loss of motor functions [5, 16]. In our study, immunizing C57BL/6 mice with MOG_35–55_ resulted in the complete paralysis of legs with clinical scoring results indicative of approximately stage 3.5. In both EAE and Tg-EAE groups, although anti-MOG antibodies implying the progression of EAE were increased, signs of EAE were not found in the Tg + EAE group. Based on this finding alone, it can be seen that *T. gondii* infection is related to the relief of EAE disease signs. To study the underlying immune mechanisms, we focused on the immune environment induced by *T. gondii* and, specifically, the increase in SOCS3 and the decrease in IL-17A. In a previous study considering these factors in innate resistance to *T. gondii*, the authors insisted that SOCS3, a target of STAT3 that limits signaling via the pleiotropic cytokine IL-6, is upregulated in response to infection but is dispensable for the immune-inhibitory effects of *T. gondii*, suggesting a critical role for SOCS3 in suppressing IL-6 signals and promoting immune responses to control infection [22]. Despite this, subsequent studies have not been performed to test its relevance to EAE.

Our results showed that the immune environment caused by *T. gondii* infection was consistent with the mechanism underlying the inhibition of EAE. For example, the following factors observed in the Tg + EAE group suggested the relief of EAE signs compared to those observed in the EAE group: decreases in cell infiltration (CD4^+^ and CD8^+^ T cells, microglia, and astrocytes), demyelination, T cell–activating cytokines and chemokines, Th17 cell lineage–associated cytokines (IL-23, IL-17A, GM-CSF), IL-6/JAK signaling molecules, phosphorylated STAT3, IL-17A transcriptional factors, JAMs, and factors involved in the breakdown of the BBB (MMPs), as well as increases in IL-27, SOCS3, Treg lineage factors (TGF-β, FoxP3, and CD25), and tight junction stability factors (Claudin-5 and β-catenin). Above all, double positive cells co-stained with both CD4^+^ and IL-17A antibodies, indicative of a Th17 cell lineage, were decreased to the same level in the control and Tg + EAE groups. In particular, the increase in Treg lineage–associated factors in the Tg + EAE group is meaningful due to the importance of the balance between Th17 and Treg cells with respect to the control of autoimmune disease [11]. In terms of *T. gondii* regulating host immunity, chronic infection especially decreased the gene expression of *IL-17A*, *IL-17R*, *IL-21*, IL-21 receptor (*IL-21R*), *IL-23*, and *IL-23R* but highly increased the gene expression of *SOCS3*. These characteristics of the immune environment comprise a unique immunomodulatory phenomenon associated with the response of *T. gondii* to host immunity. However, among the characteristics of IL-6 regulated by SOCS3, classic IL-6 signaling is responsible for the anti-inflammatory properties of IL-6, whereas trans-signaling is responsible for the pro-inflammatory actions of IL-6 [18]. As a result, dysregulation of the IL-6 axis can lead to the development of several disease states [18]. As mentioned, *T. gondii* infection leads to the suppression of anti-inflammatory IL-6 signals by SOCS3 to control infection [22]. However, uncontrolled trans-signaling via IL-6 contributes to the development of various autoimmune diseases [15, 17, 18]. During chronic infection, *T. gondii* forms an immune environment with slightly increased gene expression of *IL-6*, *JAK-1*, and *JAK-2*, which are involved in a feedback loop with STAT3. Since these signaling molecules are largely increased with the induction of EAE, the immune environment with upregulated SOCS3, induced by *T. gondii* infection, can decrease excessive IL-6–, JAK-1–, and JAK-2–mediated signals to some extent. In other words, the inhibition of IL-6 signaling pathway by SOCS3 would suppress Th17 cell differentiation [1, 17, 19]. One main role of SOCS3 results from its binding to both the JAKs, especially JAK-1 and JAK-2, and cytokine receptors, which results in the inhibition of STAT3 activation [1, 17]. The activation of STAT3 corresponds to the onset of CNS myelination, and with the development of EAE, the loss of STAT3 in CD4^+^ T cells promotes resistance to CNS inflammation [2]. Since STAT3 is required for the production of IL-17, a hallmark cytokine of the Th17 lineage, and for T cell trafficking into the CNS, the decrease in STAT3 phosphorylation and IL-17A production observed in the present study could be important to inhibit pathological autoreactive intracerebral Th17 responses and further to induce therapeutic EAE responses [3, 9, 23].

The possibility that the immune environment formed by *T. gondii* infection helps with the management of EAE can be confirmed by the lack of BBB impairment in the Tg + EAE group. Because IL-17A activates endothelial cells and the breakdown of BBB tight junctions, the reduction in IL-17A induced by *T. gondii* infection would increase BBB integrity even under conditions of EAE induction. Th17 cells highly express CCR6, and its ligand CCL20 is constitutively expressed in epithelial cells in the choroid plexus [16]. During *T. gondii* infection, the BBB acts as a selective barrier for the entry of *T. gondii* in the CNS [13]. The BBB is composed of endothelial cells that line microvessels in the brain. After *T. gondii* infection, tachyzoite forms of *T. gondii* enter the CNS via paracellular and transcellular crossing, as well as infected immune cell crossing, the so-called “Trojan horse” mechanism [13]. In this study, molecular factors such as Claudin-5, JAMs, and MMPs, related to BBB impairment, did not change during *T. gondii* infection based on our microarray data (data not shown). It is thus suggested that the “Trojan horse” mechanism for *T. gondii* entry into the CNS does not induce the functional destruction of BBB integrity. This molecular background might be helpful to maintain BBB integrity even with the development of EAE in our study. The importance of BBB integrity is related to clinical relapses mediated by inflammatory cell infiltration in the CNS [5]. Regarding the relationship between *T. gondii* infection and EAE management, the immune environment induced by *T. gondii* infection suggests the prevention of EAE pathogenicity based on multiple molecular mechanisms involving SOCS3, JAK/STAT signaling, and BBB integrity. As a result, this was suggested to block the infiltration of autoreactive T cells, Th17 cells, and Th1 T cells across the BBB and simultaneously reduce pathological inflammatory autoimmune responses in the CNS. Moreover, ME49 strain of *T. gondii* used in our study did not induce a peripheral inflammatory immune response because the pathogen migrates into the brain after infection [24]. Accordingly, the results of this study suggest that the role of toxoplasmosis in the alleviation of EAE pathology can be interpreted with reference to the immune regulation induced in the brain.

Recently, IL-17A–targeted treatment for the attenuation of autoimmune disease has been achieved by IL-17–blocking antibodies or an IL-17R antagonist [8]. For example, secukinumab, which selectively inhibits IL-17A, has now been approved for the treatment of moderate-to-severe plaque psoriasis, ankylosing spondylitis, and psoriatic arthritis. In addition, secukinumab treatment has resulted in signs of improvement in the rate of new gadolinium-enhanced lesions in MS patients [9]. Regarding other drugs in clinical trials, ixekizumab is an anti-IL-17A mAb and brodalumab is an anti-IL-17RA mAb [8]. In addition, our novel findings could encourage further clinical studies of the IL-17A pathway in MS, and for example, potential combination therapies based on molecules identified from *T. gondii* that modulate IL-17A. However, unfortunately, this study was unable to identify a single molecule that inhibits IL-17, derived from *T. gondii*. Because of this, such combination treatments cannot be realized immediately, but our study suggests the new discovery that parasites such as *T. gondii* can alleviate EAE. In conclusion, the alleviating effect of *T. gondii* on EAE development is caused not by a single agent from *T. gondii* but rather the immune regulatory mechanism of this organism that regulates the host’s immunity during the host–parasite relationship. Although this is a contextual immune characteristic of *T. gondii* infection in the brain, this is the first report to show that *T. gondii* infections can alleviate EAE.

## Conclusion

This study suggests that the alleviation of EAE after *T. gondii* infection is regulated in a SOCS3/STAT3/IL-17A/blood–brain barrier (BBB) integrity–dependent manner. Although parasite infection would not be permitted for MS treatment, this study is the first to experimentally demonstrate that infection immunity to *T. gondii* can alleviate the progression of the autoimmune disease MS. Our study provides a new promising approach for MS therapy through specific immune modulation based on CNS infection by the parasite *T. gondii*.

## Supplementary Information

ESM 1 (DOCX 18 kb)

ESM 2 (PDF 1224 kb)

ESM 3 (PDF 1224 kb)

ESM 4 (PNG 930 kb)

High Resolution Image (TIF 2753 kb)

ESM 5 (PNG 1660 kb)

High Resolution Image (TIF 5089 kb)

ESM 6 (PNG 1643 kb)

High Resolution Image (TIF 4930 kb)

ESM 7 (PNG 1716 kb)

High Resolution Image (TIF 3938 kb)

ESM 8 (PNG 870 kb)

High Resolution Image (TIF 2593 kb)

ESM 9 (PNG 1779 kb)

High Resolution Image (TIF 5451 kb)

ESM 10 (PNG 1107 kb)

High Resolution Image (TIF 3361 kb)

ESM 11 (PNG 1223 kb)

High Resolution Image (TIF 3574 kb)

## Abbreviations

BATF: basic leucine zipper ATF-like transcription factor
BBB: blood–brain barrier
CCR: CC chemokine receptors
CLTA-4: cytotoxic T lymphocyte–associated gene
CXCR: CXC chemokine receptors
EAE: experimental autoimmune encephalomyelitis
FI: fluorescence intensity
Foxp3: forkhead box P3
GFAP: glial fibrillary acidic protein
GITR: glucocorticoid-induced TNFR family-related gene
GM-CSF: granulocyte–macrophage colony-stimulating factor
H&E: hematoxylin and eosin
Iba-1: ionized calcium–binding adapter molecule 1
IL-12rβ2: interleukin-12 receptor, beta-2 subunit
iNOS: inducible nitric oxide synthase
JAK: Janus kinase
JAM: junctional adhesion molecule
LFB: Luxol fast blue
MMP: matrix metalloproteinases
MOG_35–55_: myelin oligodendrocyte glycoprotein 35–55
MS: multiple sclerosis
NO: nitric oxide
PTX: pertussis toxin
RORγt: RAR-related orphan receptor gamma
RUNX1: Runt-related transcription factor 1
SOCS3: suppressor of cytokine signaling 3
STAT: signal transducer and activator of transcription
T-bet: T-box transcription factor TBX21
TGF-β: transforming growth factor beta
*T. gondii*: *Toxoplasma gondii*
Th-GM: GM-CSF–producing T helper
Treg: regulatory T cells
