# Supplementary material for: Chronic Toxoplasma gondii Infection Alleviates Experimental Autoimmune Encephalomyelitis by the Immune Regulation Inducing Reduction in IL-17A/Th17 Via Upregulation of SOCS3
Source: Neurotherapeutics. 2020 Nov 17;18(1):430–47. doi: 10.1007/s13311-020-00957-9 (PMC8116467; doi:10.1007/s13311-020-00957-9)
Supplement: Supplementary file 1 — (DOCX 18 kb) [file 13311_2020_957_MOESM1_ESM.docx]

| **Clinical score** | **Clinical observation** |
| --- | --- |
| **0** | No obvious changes of motor function compared to control mice. When picked up the base of tail, the tail has tension and has no disability for movement. |
| **0.5** | When picked up the base of tail, tip of tail is limp.  the tail has tension except for the tip. |
| **1** | When picked up by base of tail, Limp tail and the whole tail drapes over finger. Hind legs are usually spread apart. |
| **1.5** | When picked up by base of tail, Limp tail and hind leg inhibition of movement and the whole tail drapes over finger. When the mouse is dropped on a wire rack, at least one hind leg falls through consistently. Walking is very slightly wobbly |
| **2** | When picked up by base of tail, Limp tail and weakness of hind legs and the legs are not spread apart, but held closer together. When the mouse is observed walking, it has a clearly apparent wobbly walk. One foot may have toes dragging, but the other leg has no apparent inhibitions of movement. |
| **2.5** | Limp tail and dragging of hind legs. Both hind legs have some movement, but both are dragging at the feet. or No movement in one leg/completely dragging one leg, but movement in the other leg |
| **3** | Limp tail and complete paralysis of hind legs or Limp tail and almost complete paralysis of hind legs or One or both hind legs are able to paddle, but neither hind leg is able to move forward of the hind hip |
| **3.5** | Limp tail and complete paralysis of hind legs. In addition to: Mouse is moving around the cage, but when placed on its side, is unable to right itself. Hind legs are together on one side of body or Mouse is moving around the cage, but the hind quarters are flat like a pancake, giving the appearance of a hump in the front quarters of the mouse. |
| **4** | Limp tail, complete hind leg and partial front leg paralysis. Mouse is minimally moving around the cage but appears alert and feeding. |
| **4.5** | Complete hind and partial front leg paralysis, no movement around the cage. Mouse has minimal movement in the front legs. The mouse barely responds to contact. |
| **5** | Mouse is spontaneously rolling in the cage (euthanasia is recommended) or Mouse is found dead due to paralysis or Mouse is euthanized due to severe paralysis. |

**Table 1. Mouse EAE scoring**

**Table 2. Primer sequence used by conventional PCR and qRT-PCR**

| **GENE** | **FORWARD** | **REVERSE** |
| --- | --- | --- |
| **SOCS3** | **5′-CGA AGG GAG GCA GAT CAA CA-3′** | **5′-TGG CTG TGT TTG GCT CCT T-3′** |
| **RORγt** | **5′-TCA CCC AGC CTT TCC CTT TC-3′** | **5′-CAG AAG CCA GGG TGG AAC TT-3′** |
| **IL-23** | **5′-TCT GCA TGC TAG CCT GGA AC-3′** | **5′-CAG ACC TTG GCG GAT CCT TT-3′** |
| **IL-17A** | **5′-ACC GCA ATG AAG ACC CTG AT-3′** | **5′-CAC ACC CAC CAG CAT CTT CT-3′** |
| **TGF-β** | **5′-CCT TCC TGC TCC TCA TGG-3′** | **5′-CGC ACA CAG CAG TTC TTC-3′** |
| **IL-10** | **5′-GCC CTT TGC TAT GGT GTC CT-3′** | **5′-GGG ATG ACA GTA GGG GAA CC-3′** |
| **IL-6** | **5′-GGA GCC CAC CAA GAA CGA TA-3′** | **5′-ACA GGT CTG TTG GGA GTG GT-3′** |
| **IL-1β** | **5′-GTG GCA GCT ACC TGT GTC TT-3′** | **5′-GGA GCC TGT AGT GCA GTT GT-3′** |
| **IL-2** | **5′-AGATGAACTTGGACCTCTGCG-3′** | **5′-AAAGTCCACCACCACAGTTGCTG -3′** |
| **T-bet** | **5′-ATTGGTTGGAGAGGAAGCGG -3′** | **5′- GCACCAGGTTCGTGACTGTA-3′** |
| **CTLA-4** | **5′-ACTGAGAGCTGTTGACACGG -3′** | **5′-ACATTCTGGCTCTGTTGGGG -3′** |
| **IL-12Rβ2** | **5′-TTTGAGGGTTCAGCAAAAAGGC -3′** | **5′- TGGTGCTTTGTGCTTGGAGT-3′** |
| **GITR** | **5′-ATCTGCAAGCACTACCCCTG -3′** | **5′-GCATTGTGGGTCTTGTTCCC -3′** |
| **TNF- α** | **5′-GTC ATT GCT CTG TGA AGG GAA TG-3′** | **5′-GAG TTG GAC CCT GAG CCA TAA TC-3′** |
| **IL-27** | **5′-TGTCCACAGCTTTGCTGAAT -3′** | **5′-GCCGAAGTGGTGGTAGCGA-3′** |
| **JAK1** | **5′-GAC CAG GCA AGA TCC AGA CAT -3′** | **5′-ACT CTC AGG CTT CAG GGA CT -3′** |
| **JAK2** | **5′-GCG ACG GGA ACA AGA TGT GA -3′** | **5′-TTG TAA GGC AGG CCA TTC CC -3′** |
| **BATF** | **5′-GGACAAGAAGGGCGATGCTA -3′** | **5′-GGG TCC AGT ACA TTG GCT CG -3′** |
| **RUNX1** | **5′- GGCAGGACGAATCACACTGA-3′** | **5′- CGA AAA CGC ACC TCT CCT GA-3′** |
| **CD69** | **5′-GCT CCA GCT ACA TCT CTC CG -3′** | **5′- CAT GGT CCT TCT GCC CTC TC-3′** |
| **CD44** | **5′- ACC TTG GCC ACC ACT CCT AAT A-3′** | **5′- GAC TGG AGT CTG TAT CCT TGC T-3′** |
| **PD-1** | **5′- GAC TGC TAC TGA AGG CGA CA-3′** | **5′-AGC ACA GCC CAA GTG AAT GA -3′** |
| **CCR5** | **5′-AGA CAT CCG TTC CCC CTA CA -3′** | **5′- GCA GGG TGC TGA CAT ACC AT-3′** |
| **CCR6** | **5′- AAGGTATCTACCCCAGGAGGGTG-3′** | **5′-GGG AGA GCA GAG GTG AAG CAA -3′** |
| **CXCR3** | **5′- GCA GCC CAA GTC CTA ACA CA-3′** | **5′- CCT GGC CCT CTG TGC TAT TT-3′** |
| **CD11b** | **5′-CCA CAC TAG CAT CAA GGG CA -3′** | **5′-GCT TCA CAC TGC CAC CGT -3′** |
| **Claudin-5** | **5′- TTT CTT CTA TGC GCA GTT GG -3′** | **5′- GCA GTT TGG TGC CTA CTT CA -3′** |
| **GAPDH** | **5′-CATGGCCTCCAAGGAGTAAG-3′** | **5′-CCTAGGCCCCTCCTGTTATT-3′** |
