# Supplementary figures and images for: Chronic Toxoplasma gondii Infection Alleviates Experimental Autoimmune Encephalomyelitis by the Immune Regulation Inducing Reduction in IL-17A/Th17 Via Upregulation of SOCS3
Source: Neurotherapeutics. 2020 Nov 17;18(1):430–47. doi: 10.1007/s13311-020-00957-9 (PMC8116467; doi:10.1007/s13311-020-00957-9)

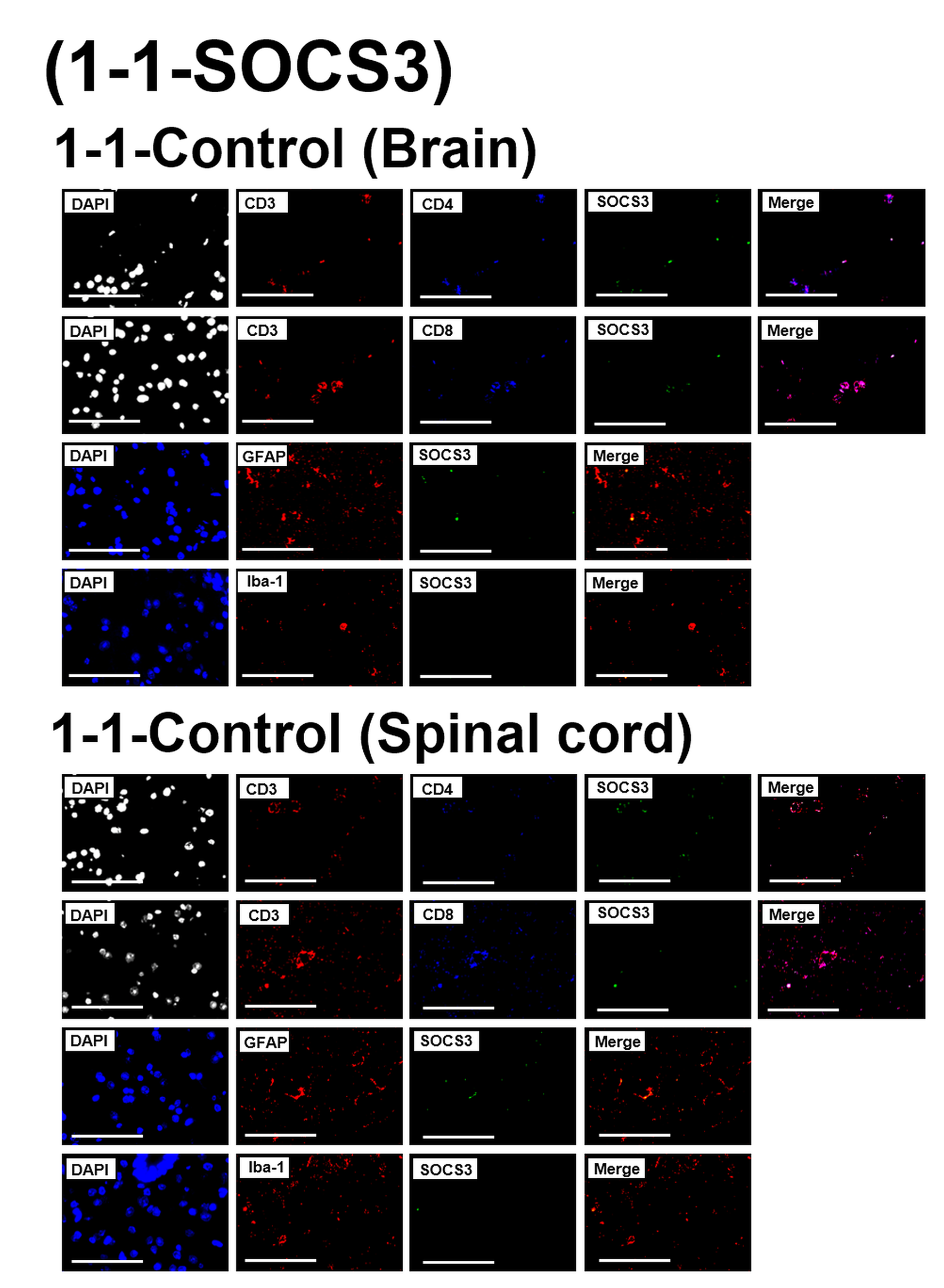

Supplement: Supplementary file 4 — (PNG 930 kb) [file 13311_2020_957_Fig11_ESM.png]

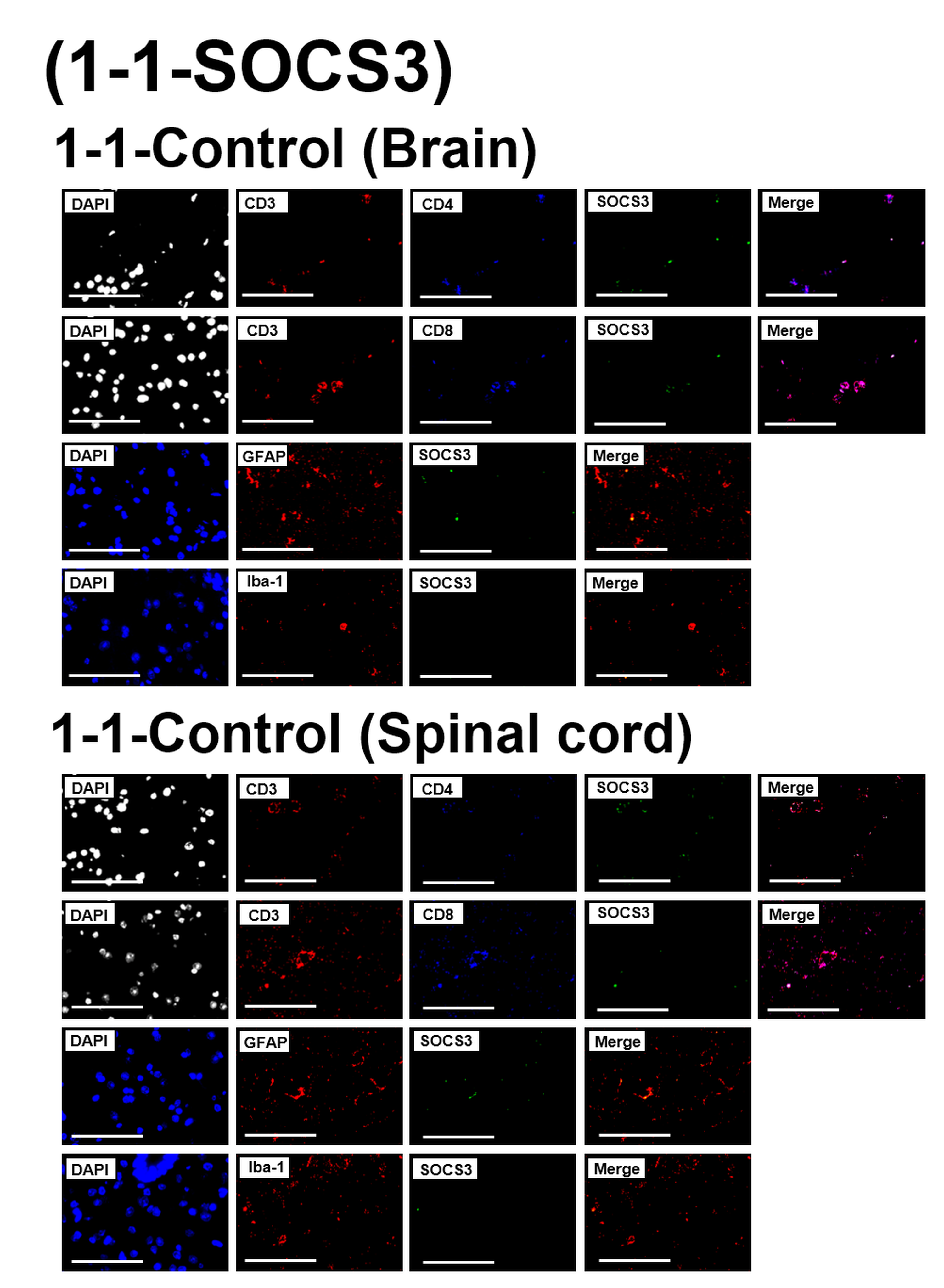

Supplement: Supplementary file 5 — High Resolution Image (TIF 2753 kb) [file 13311_2020_957_MOESM4_ESM.tif]

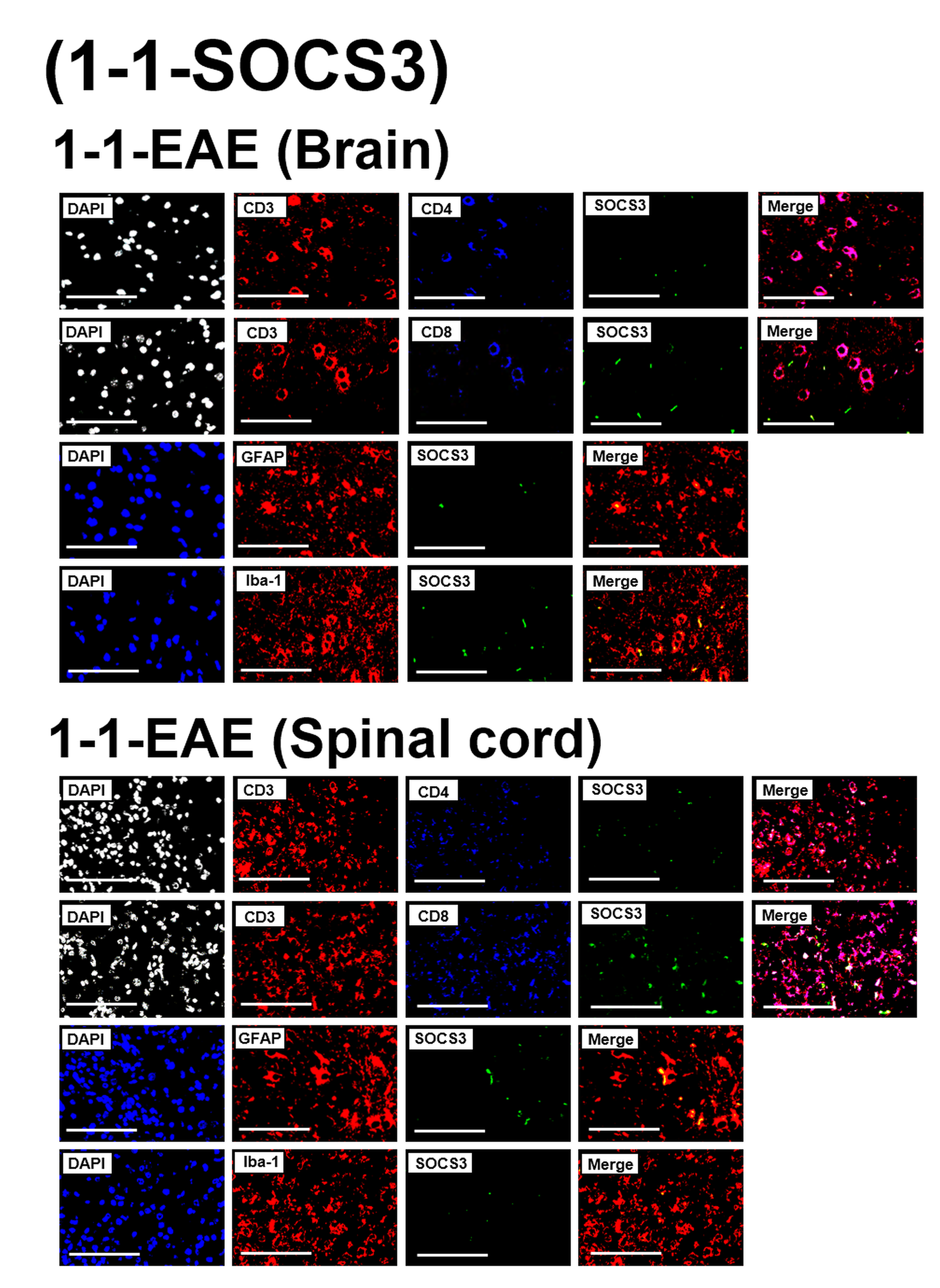

Supplement: Supplementary file 6 — (PNG 1660 kb) [file 13311_2020_957_Fig12_ESM.png]

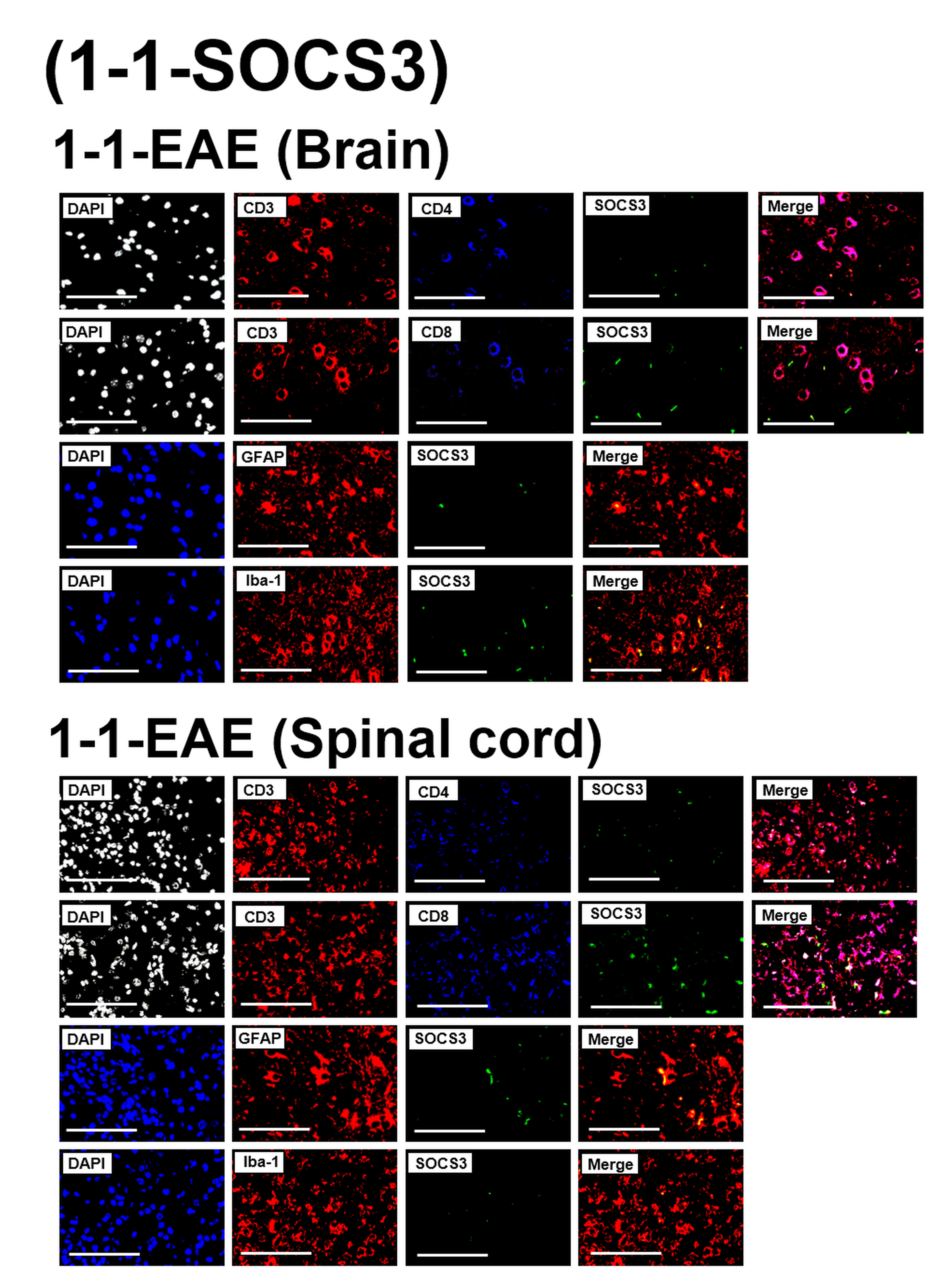

Supplement: Supplementary file 7 — High Resolution Image (TIF 5089 kb) [file 13311_2020_957_MOESM5_ESM.tif]

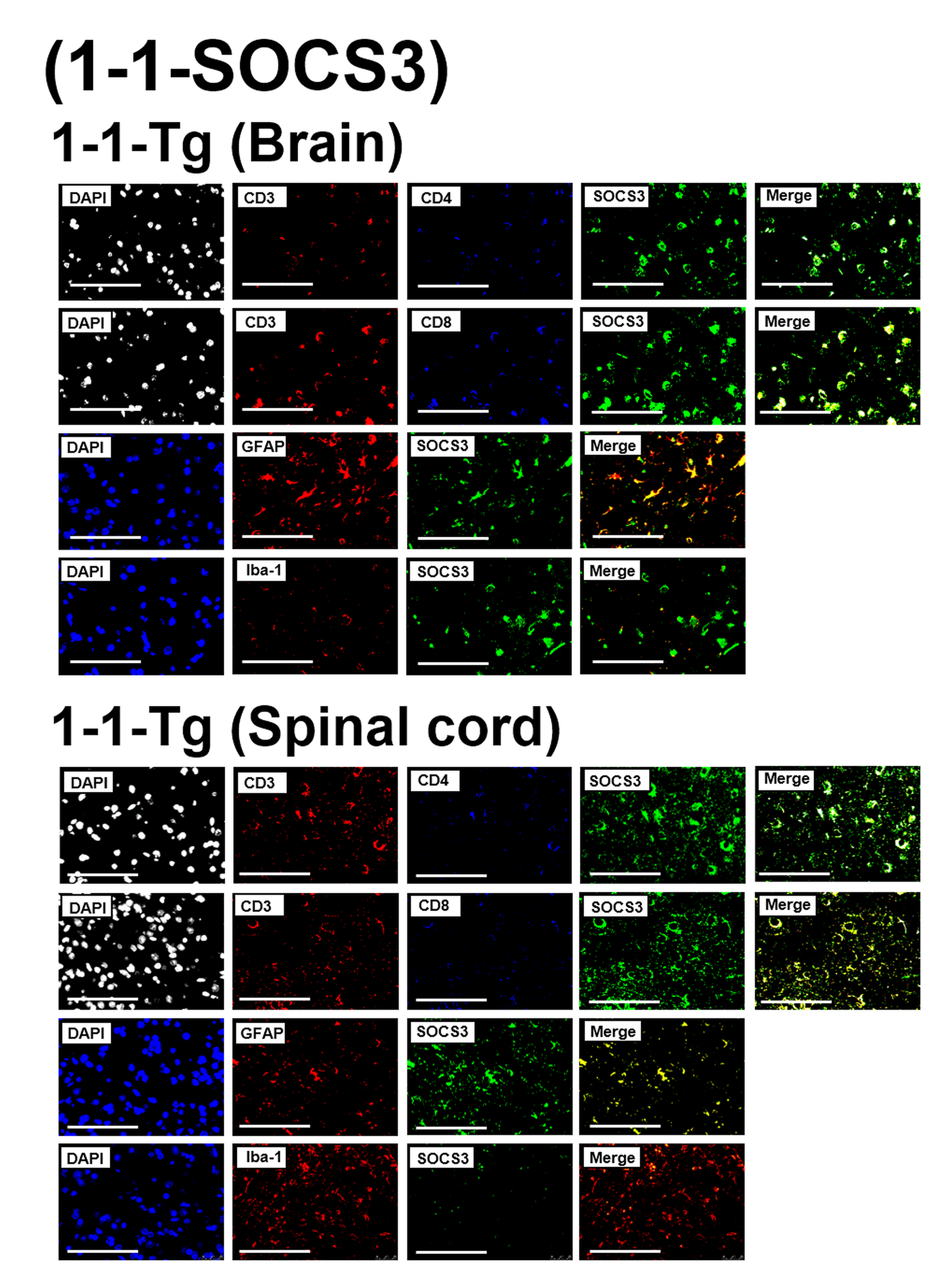

Supplement: Supplementary file 8 — (PNG 1643 kb) [file 13311_2020_957_Fig13_ESM.png]

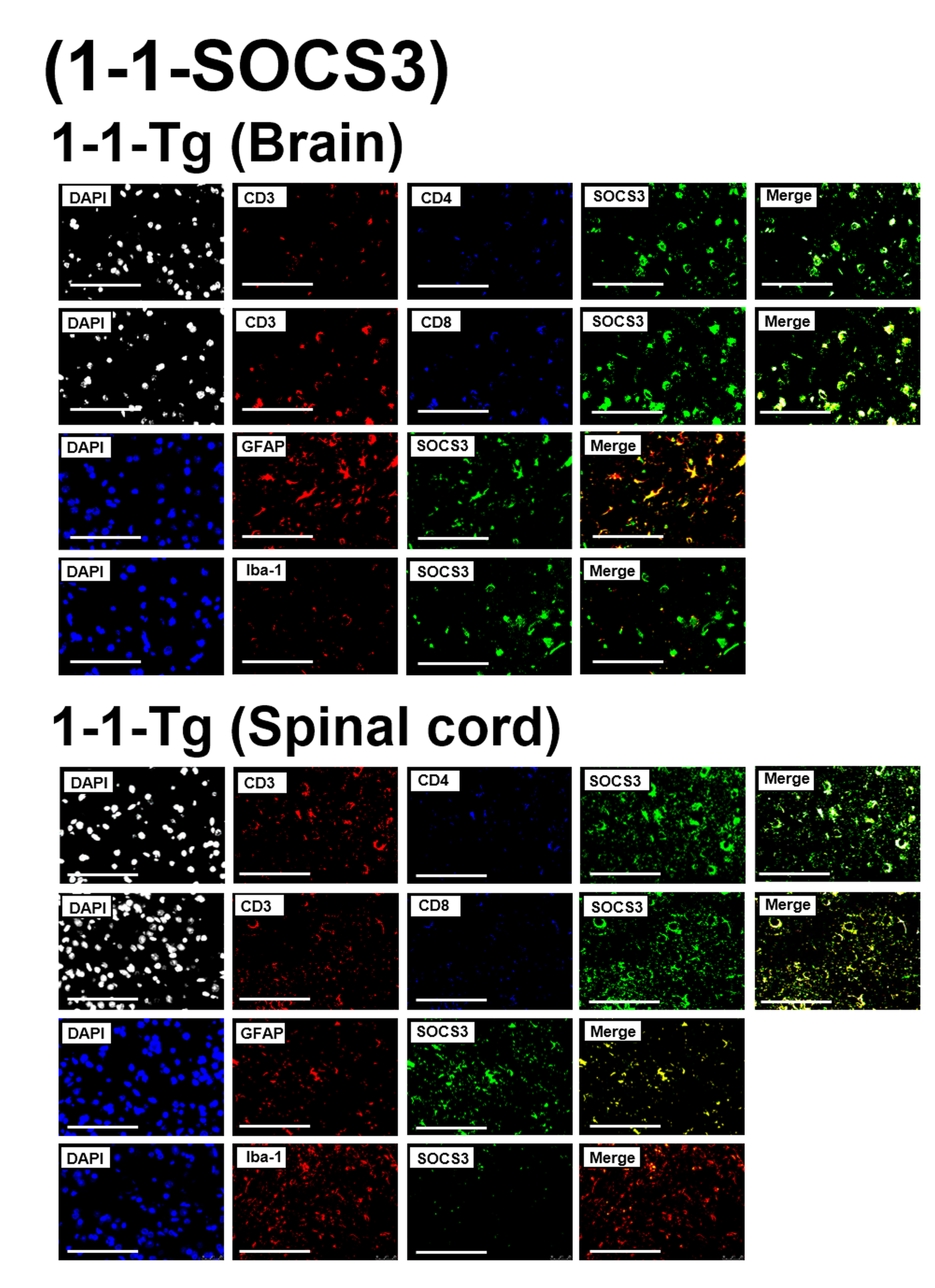

Supplement: Supplementary file 9 — High Resolution Image (TIF 4930 kb) [file 13311_2020_957_MOESM6_ESM.tif]

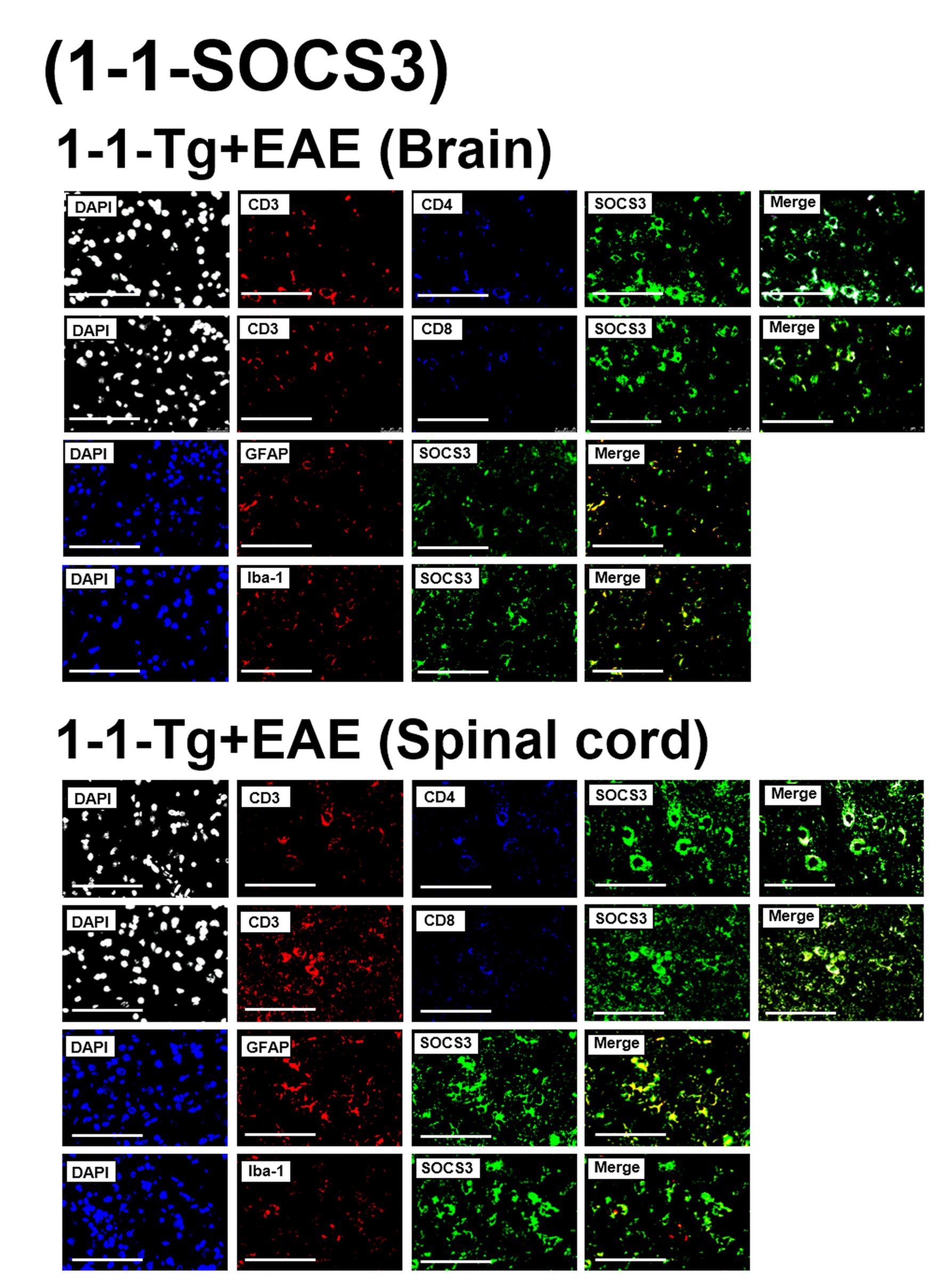

Supplement: Supplementary file 10 — (PNG 1716 kb) [file 13311_2020_957_Fig14_ESM.png]

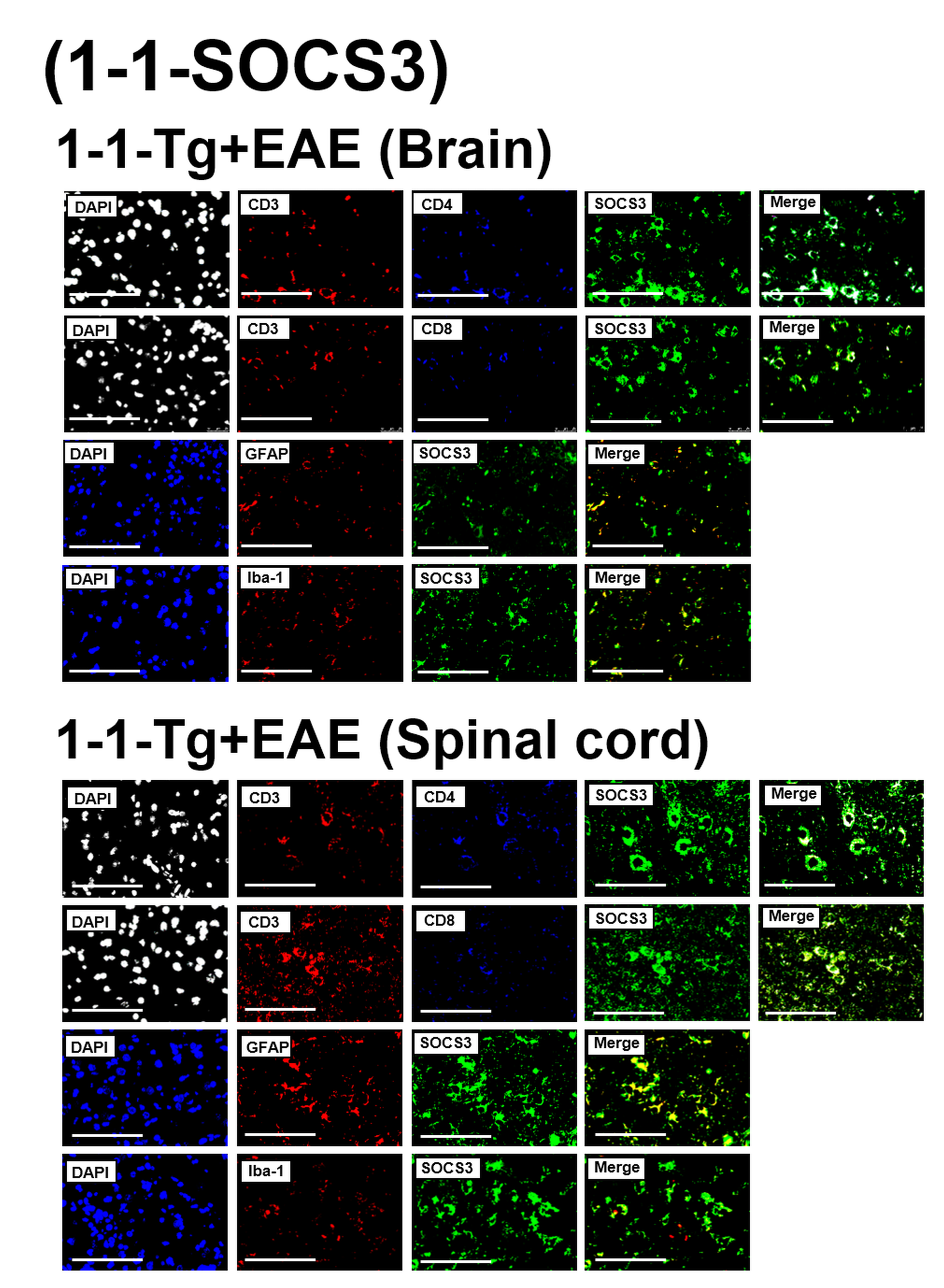

Supplement: Supplementary file 11 — High Resolution Image (TIF 3938 kb) [file 13311_2020_957_MOESM7_ESM.tif]

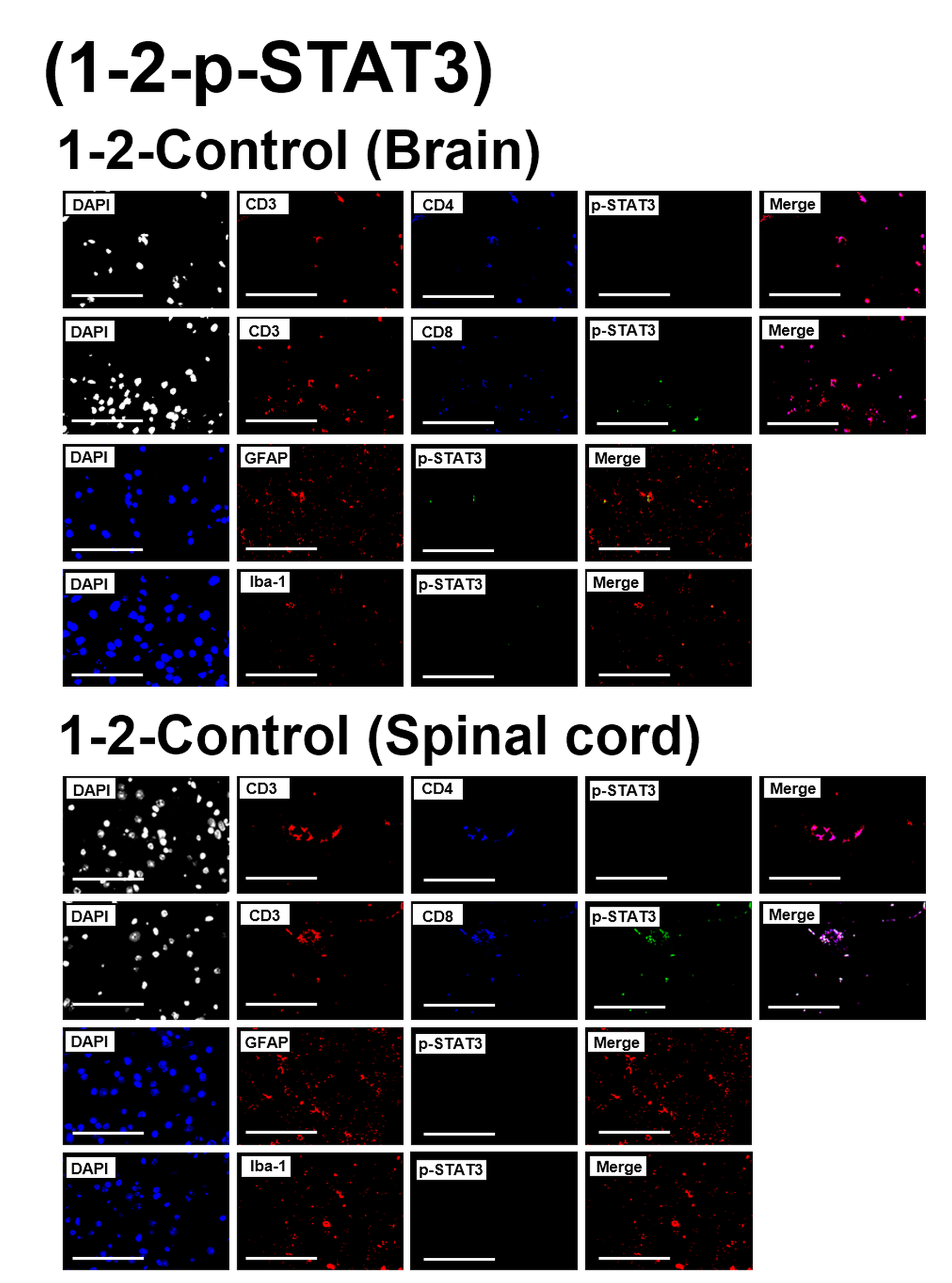

Supplement: Supplementary file 12 — (PNG 870 kb) [file 13311_2020_957_Fig15_ESM.png]

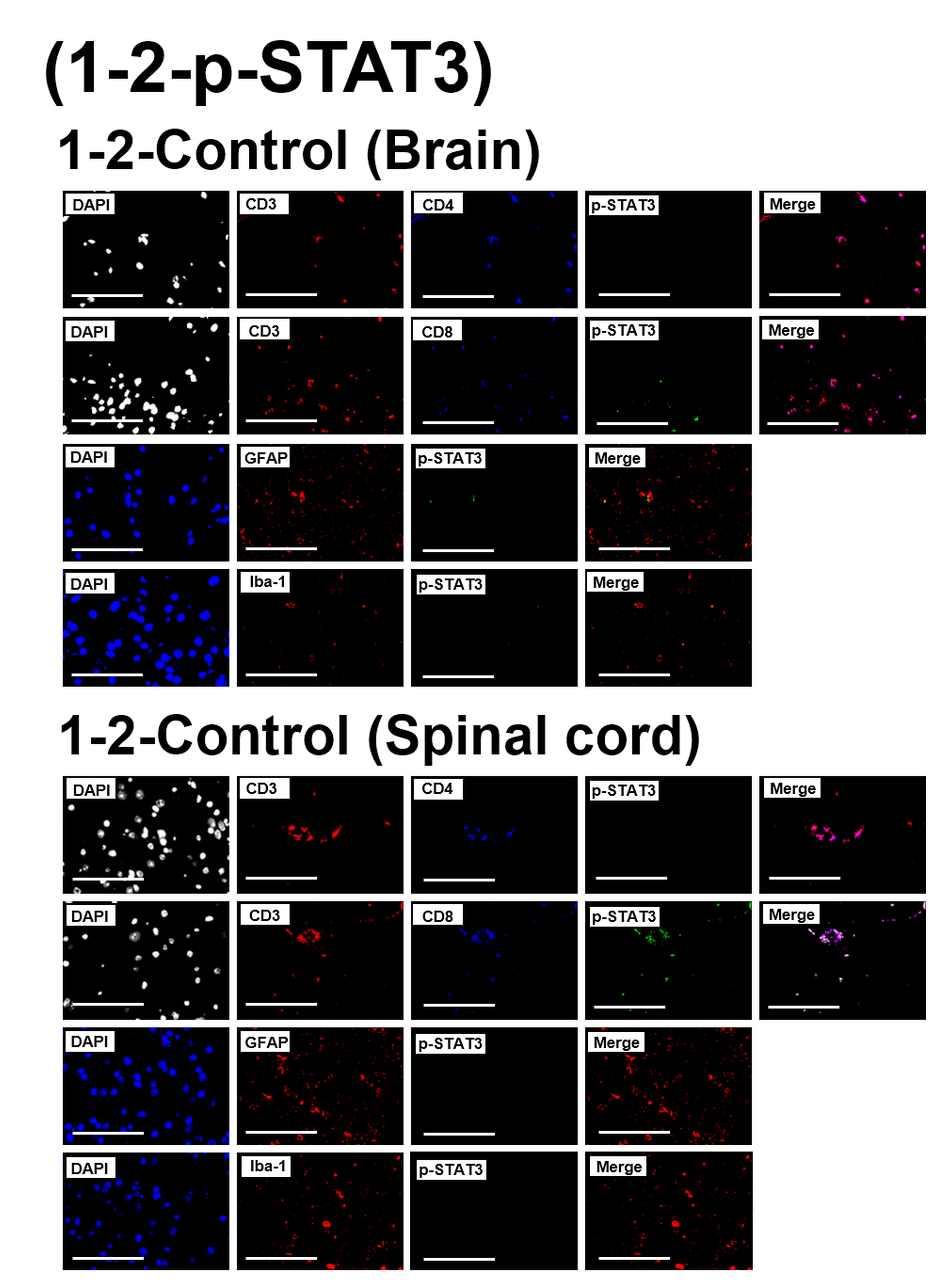

Supplement: Supplementary file 13 — High Resolution Image (TIF 2593 kb) [file 13311_2020_957_MOESM8_ESM.tif]

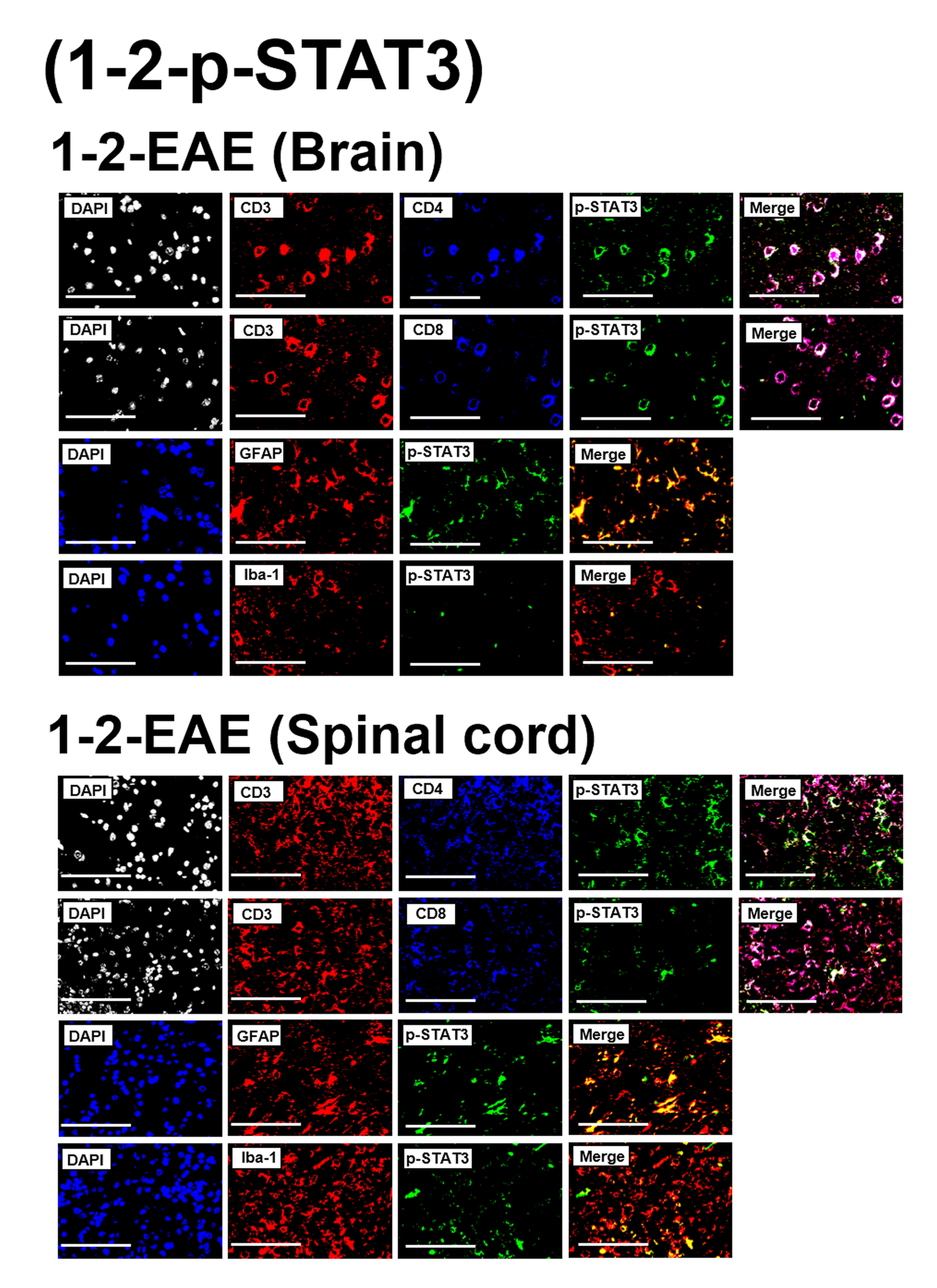

Supplement: Supplementary file 14 — (PNG 1779 kb) [file 13311_2020_957_Fig16_ESM.png]

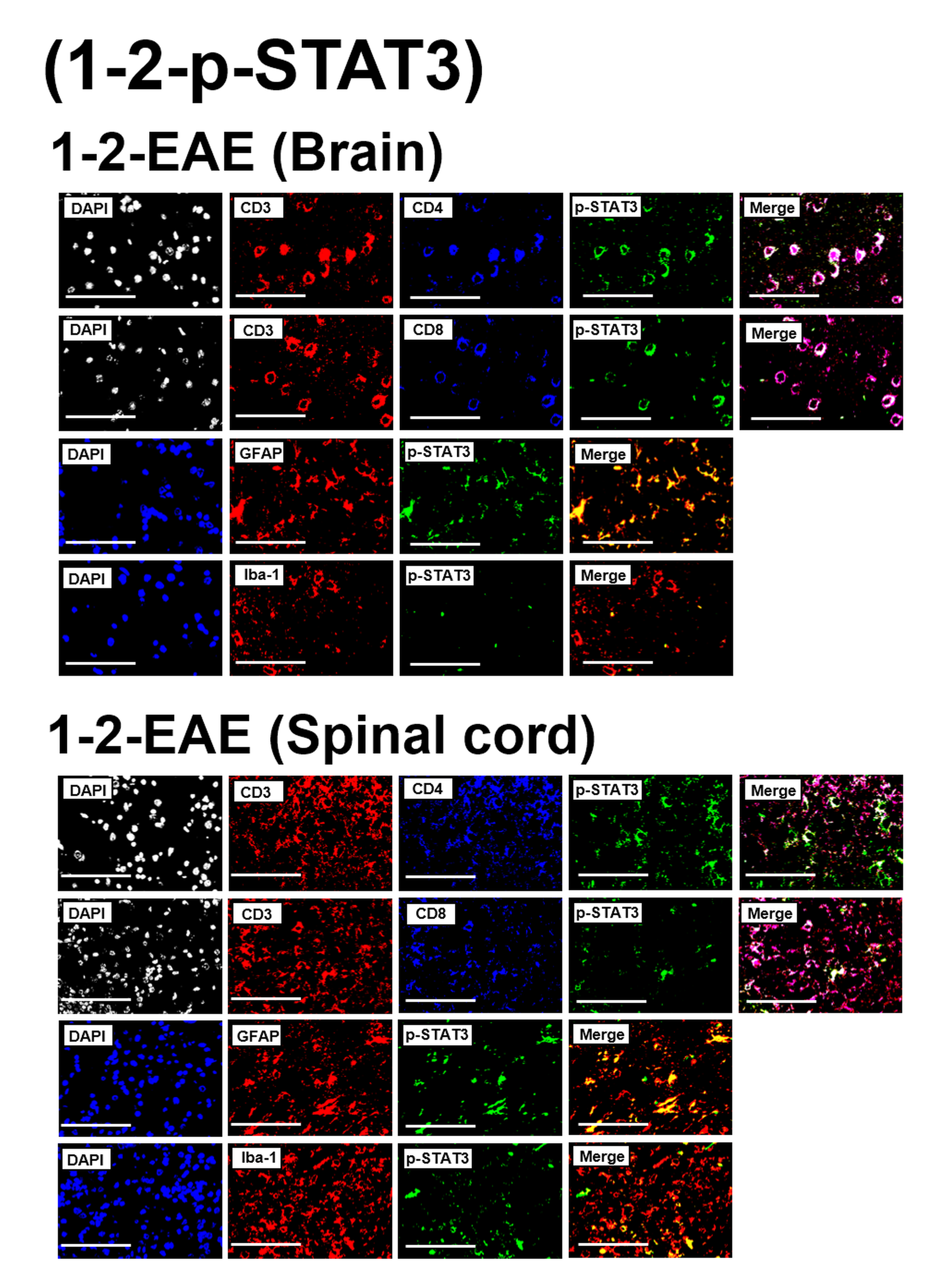

Supplement: Supplementary file 15 — High Resolution Image (TIF 5451 kb) [file 13311_2020_957_MOESM9_ESM.tif]

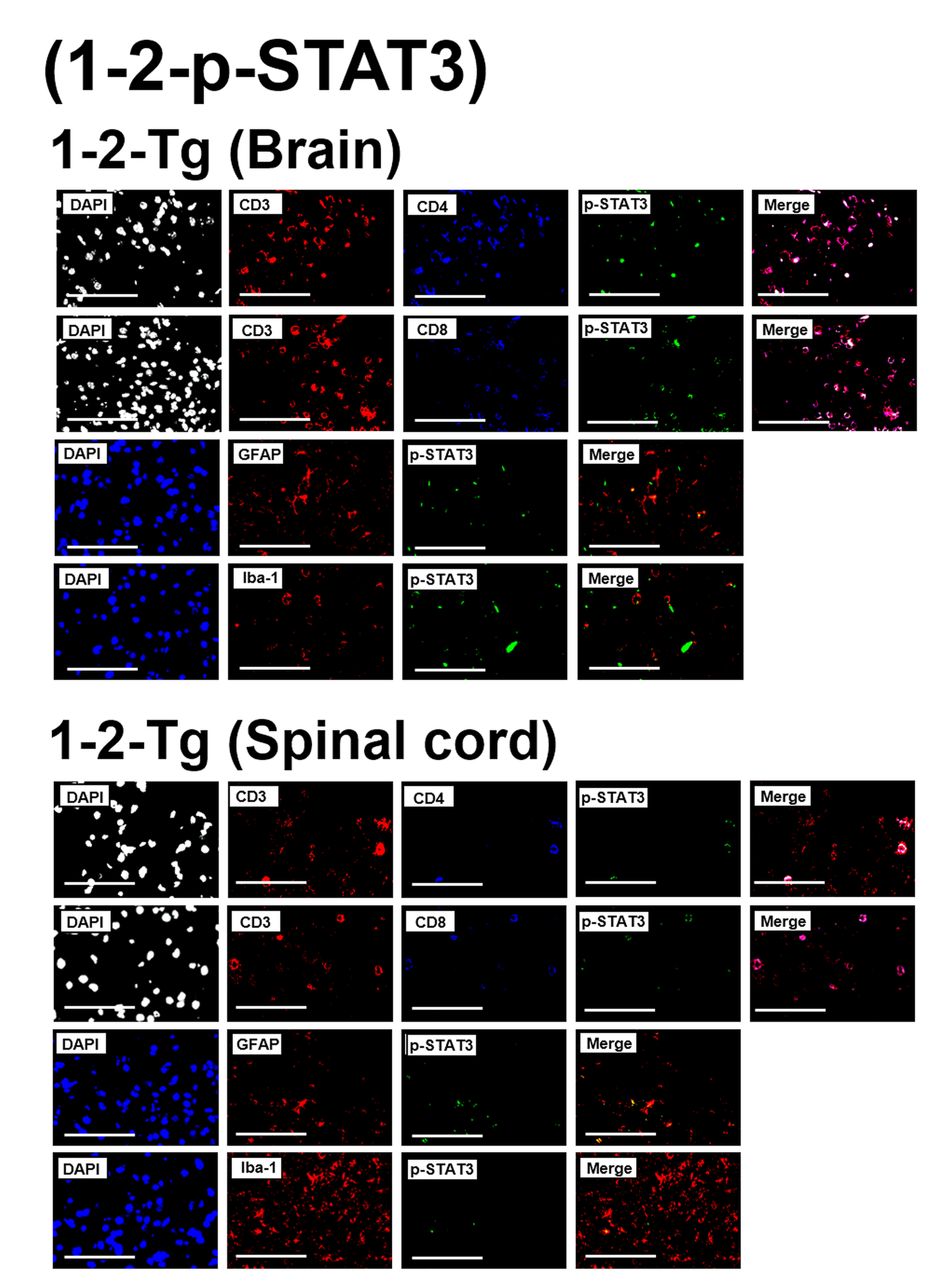

Supplement: Supplementary file 16 — (PNG 1107 kb) [file 13311_2020_957_Fig17_ESM.png]

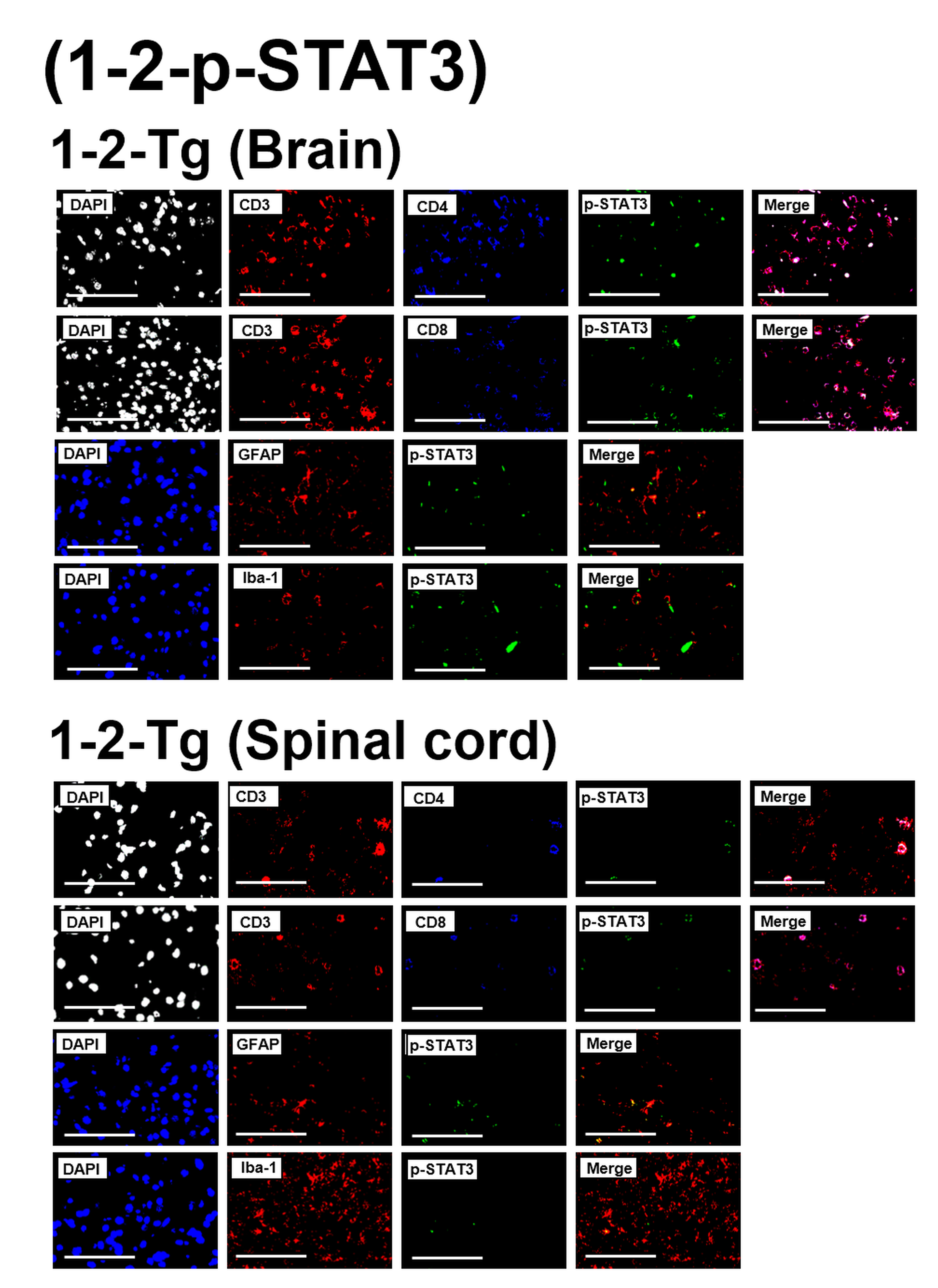

Supplement: Supplementary file 17 — High Resolution Image (TIF 3361 kb) [file 13311_2020_957_MOESM10_ESM.tif]

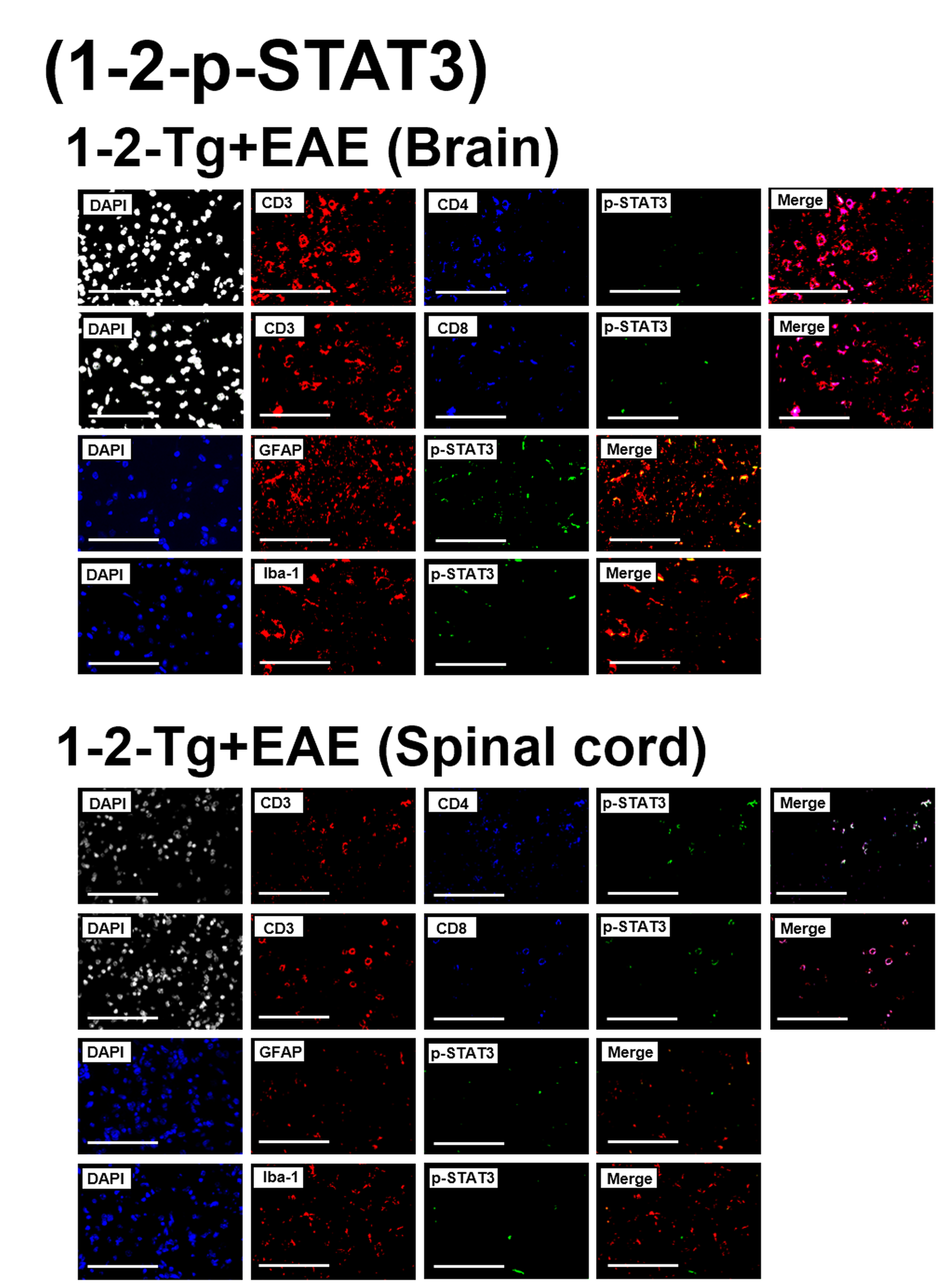

Supplement: Supplementary file 18 — (PNG 1223 kb) [file 13311_2020_957_Fig18_ESM.png]

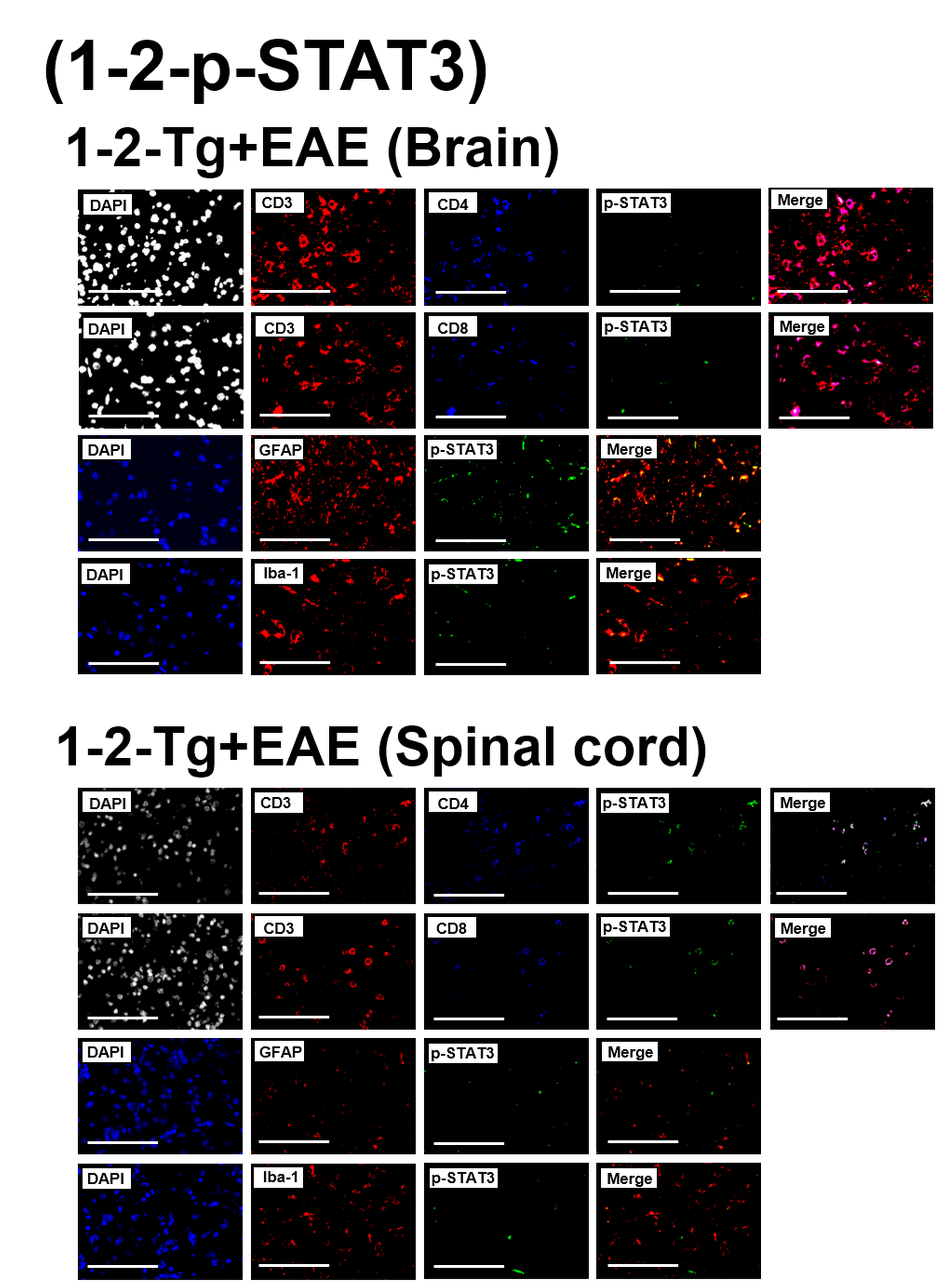

Supplement: Supplementary file 19 — High Resolution Image (TIF 3574 kb) [file 13311_2020_957_MOESM11_ESM.tif]
